# Overexpression of a novel gene (*Pt2015*) endows the commercial diatom *Phaeodactylum tricornutum* high lipid content and grazing resistance

**DOI:** 10.1186/s13068-022-02221-y

**Published:** 2022-11-26

**Authors:** Shan Gao, Lu Zhou, Wenting Yang, Lijun Wang, Xuehua Liu, Yingchun Gong, Qiang Hu, Guangce Wang

**Affiliations:** 1 CAS and Shandong Province Key Laboratory of Experimental Marine Biology, Center for Ocean Mega‑Science, Institute of Oceanology, Chinese Academy of Sciences, Qingdao, China; 2 Laboratory for Marine Biology and Biotechnology, Qingdao National Laboratory for Marine Science and Technology, Qingdao, China; 3 College of Earth Sciences, University of Chinese Academy of Sciences, Beijing, 100049 China; 4 College of Life Science, Qingdao Agricultural University, Qingdao, China; 5 Center for Microalgal Biotechnology and Biofuels, Institute of Hydrobiology, Chinese Academy of Sciences, Wuhan, 430072 China; 6 CAS Key Laboratory of Quantitative Engineering Biology, Shenzhen Institute of Synthetic Biology, Shenzhen Institute of Advanced Technology, Chinese Academy of Sciences, Shenzhen, 518055 China; 7 Faculty of Synthetic Biology, Shenzhen Institute of Advanced Technology, Chinese Academy of Sciences, Shenzhen, 518055 China

**Keywords:** Diatom, Gene manipulation, Lipid, Morphotype, *Phaeodactylum tricornutum*

## Abstract

**Background:**

The marine diatom *Phaeodactylum tricornutum* is a commercially viable species due to its bioactive substances and lipid productivity. Increasing attention has been paid to the isolation or genetic modification of species or strains with a rapid growth rate and large quantities of lipids. Furthermore, contamination of microzooplankton has been one of the major constraints in *P. tricornutum* large-scale cultivation, which adversely affects growth and greatly impedes the course of biomass production industrialization.

**Results:**

Here, based on our previous transcriptomics of *P. tricornutum*, we found a novel gene (ID: 7202015, hereafter called *Pt2015*) which affects morphotype of *P. tricornutum*. Pt2015 protein is located in the plastid, which is highly homologous to part of the sequences of exosome component. The morphotype of the *Pt2015* knockout strain (termed 2015KO) using CRISPR/Cas9 method is fusiform, but the *Pt2015* overexpression strain (termed oeT) demonstrates a majority triradiate morphotype (approximately 95%) which is stable and has been cultured for more than 200 generations. In addition, the oeT strain demonstrated a similar growth rate to the WT and simultaneously accumulated larger lipids droplets that increased by approximately 30% compared to that of the WT. More importantly, the grazing rate of the amoebae cultured in the oeT strain significantly decreased in comparison with that cultured in WT, suggesting that the oeT can effectively avoid being eaten by microzooplankton.

**Conclusions:**

Therefore, the oeT strain not only improves our understanding of morphotype conversion in diatoms but also demonstrates potential applications for large-scale cultivation of *P. tricornutum*.

**Supplementary Information:**

The online version contains supplementary material available at 10.1186/s13068-022-02221-y.

## Background

Diatoms are unicellular eukaryotic microalgae responsible for approximately 20% of the primary production on Earth and are the dominant primary producers in the ocean [1]. They are highly abundant and diverse, with estimates of more than 100,000 species [2]. Diatoms have been exploited on a commercial scale for decades due to their bioactive substances, silica biomineralization, and lipid productivity [3, 4]. *Phaeodactylum tricornutum* is one of the most extensively studied diatom species. Its genome has been sequenced [5], and genetic manipulation, including gene silencing and CRISPR/Cas9, has been proven to be efficient in this species [6–9]. These resources and tools will help investigate gene function and decipher cellular processes in diatoms. Therefore, *P. tricornutum* has been a model for studying diatom biology at the molecular level. Additionally, due to its robust growth, a well-characterized genome, and a tailored molecular toolbox, *P. tricornutum* has become a vital microalgal candidate for a cell chassis and plays an important role in synthetic biology.

*P. tricornutum* is also a commercially important species that is widely used in aquaculture, and is rich in active substances, such as eicosapentaenoic acid and fucoxanthin [10, 11]. Furthermore, *P. tricornutum* synthesizes and stores neutral lipids (mainly triglycerides) in lipid droplets, which can be converted to biodiesel, and thus, it has potential to be a useful biofuel [11]. A general trend toward lipid accumulation in response to unfavorable environmental or stressful conditions has been observed in *P. tricornutum* [11]. Nitrogen and phosphorus limitations have been successfully utilized to accumulate lipids in *P. tricornutum* [12–14]. Nevertheless, unfavorable environmental or stressful conditions, including nutrient limitations, profoundly decrease growth rate and biomass production [11]. Therefore, increasing attention has been paid to the isolation or genetic modification of species or strains that can grow rapidly and simultaneously accumulate large quantities of lipids.

Due to its huge commercial value, large-scale cultivation of *P. tricornutum* also has gained increasing attention [15]. In fact, *P. tricornutum* is a successful photoautotroph, which has the capacity to grow in seawater and to utilize carbon dioxide as a feedstock. Thus, low cost is a key advantage of large-scale cultivation of *P. tricornutum*. However, contamination of algivorous microzooplankton has been one of the main constraints in *P. tricornutum* large-scale cultivation, which adversely affects growth and greatly impedes the course of biomass production industrialization [16–18]. Many control strategies, including physical, chemical, and biological methods, have been proposed to overcome the challenges of microzooplankton contamination [17, 19–21]. However, these methods are costly and time consuming. Therefore, isolating a new strain with grazing resistance represents an important strategy for overcoming zooplankton contamination [22].

In fact, in natural aquatic systems, changing the morphological features, such as size, cell shape, or geometry of phytoplankton, is the most obvious way to reduce grazing pressure from zooplankton [23–27]. In response to different conditions, *P. tricornutum* can display at least three distinct morphotypes: fusiform, oval, and triradiate [28–32]. The fusiform morphotype is the most frequent and stable type in cell cultures [30]. The oval morphotype tends to be benthic, having a lower growth rate and higher sedimentation rate [33, 34]. The triradiate morphotype isolated from natural conditions is favored in an unstressed planktonic environment, which possesses specific geometry with three distal arms [30]. Therefore, the triradiate cells of *P. tricornutum* may be one of the best morphotypes to withstand grazing pressure.

Our previous transcriptomics of *P. tricornutum* treated with fluctuating light conditions suggested that thousands of genes in *P. tricornutum* were related to fluctuating light conditions. Among these genes, we chose several genes including *Pt2015* (ID: 7202015) in order to investigate their roles under fluctuating light. Unexpectedly, we found that *Pt2015* is not close to fluctuating light condition, but it affects morphotypes conversion and lipid metabolisms in *P. tricornutum*. Moreover, *Pt2015* encodes an unknown protein, and to date, there is no knowledge regarding the function of this gene in diatoms. Therefore, to investigate the function of this gene, we constructed *Pt2015* knockout and overexpression strains, respectively. We found that overexpression of *Pt2015* caused a *P. tricornutum* triradiate strain called overexpression triradiate (oeT), in which the abundance of triradiate cells was approximately 95% under normal growth condition. The oeT strain was stable, making it insensitive to hyposalinity and low temperature. More importantly, the oeT strain showed a similar growth rate to the WT, accumulated larger lipids droplets, and withstood grazing pressure, demonstrating potential applications for biofuel production.

## Materials and methods

### Cell cultures

Axenic *P. tricornutum* cells were obtained from the Institute of Hydrobiology, Chinese Academy of Sciences, and grown at 20 °C in a photoperiod 16 h:8 h, light:dark. Cells were grown under 80 μmol photons m^−2^ s^−1^ and collected during the exponential growth phase.

### Phylogenetic analysis and prediction of Pt2015 localization in *P. tricornutum*

**Fig. 1:**
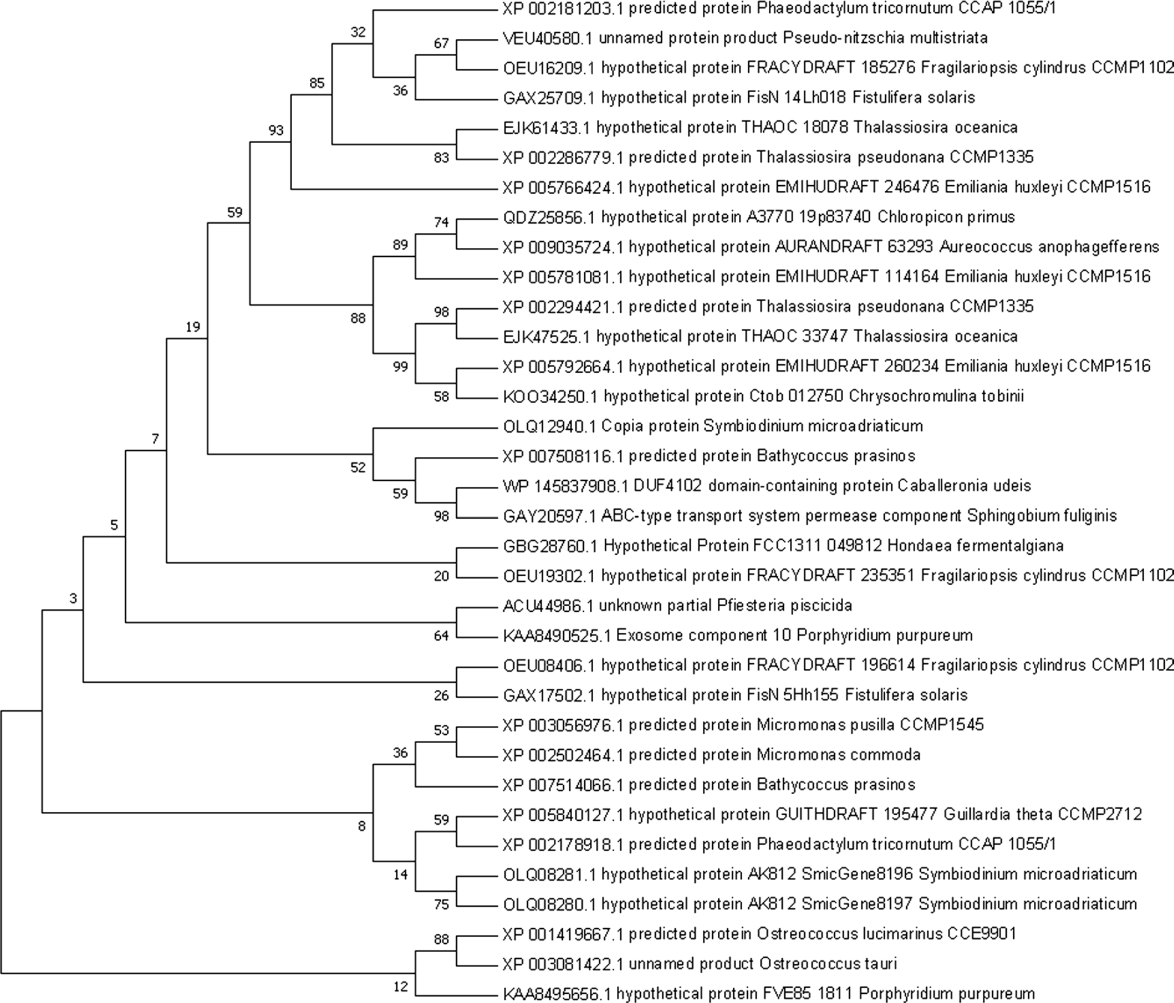
Phylogenetic analysis of Pt2015 proteins. In total, 34 protein sequences were used to perform phylogenetic analysis using the MEGA 7.0 platform and the neighbor-joining method. The percentage of replicate trees in which the associated taxa cluster together in the bootstrap test (500 replicates) is shown next to the branches

Phylogenetic analysis of Pt2015 proteins was performed using the MEGA v.7.0 platform using the neighbor-joining method. In total, 34 protein sequences were used to perform the phylogenetic analysis. The percentage of replicate trees in which the associated taxa clustered together in the bootstrap test (500 replicates) was shown next to the branches in Fig. 1. Moreover, a comparison of the conserved regions in the Pt2015 protein from diatoms, chrysophytes, dinoflagellates, and other red-tide algae was performed using the MEGA v.7.0 platform. In addition, the cloned Pt2015 sequences were analyzed for the presence of N-terminal signal peptides and cleavage sites using the SignalP v.4.1 Server (www.cbs.dtu.dk/services/SignalP/). The TMHMM Server v.2.0 (www.cbs.dtu.dk/services/TMHMM/) was used to predict transmembrane domains.

### Construction of *Pt2015* knockout strains using CRISPR/Cas9

*Pt2015* knockout strains were constructed according to a previous method in which the Cas9 system was delivered into *P. tricornutum* via conjugation of plasmids from a bacterial donor cell [7, 8]. Briefly, three guide RNAs (gRNAs) targeting *Pt2015* (Additional file 1: Table S1) were designed on the website (http://crispor.tefor.net/). To generate gRNA cloning inserts, the designed oligonucleotide corresponding to the predicted gRNA binding site and the complementary oligonucleotide were ordered, and then phosphorylated and annealed. Using Golden Gate assembly (New England Biolabs), cloned gRNA was inserted into the pPtGE35 plasmid obtained from Addgene (107999). The product from the Golden Gate reaction was transformed into Epi300 *E. coli* cells using heat shock. Then, the properly cloned gRNA plasmid was transformed into DH10B *E. coli* cells containing the pTA-Mob plasmid [35] obtained from Dr. Rahmi Lale (Norwegian University of Science and Technology, Trondheim, Norway).

Subsequently, according to the published protocol [8], the pPtGE35 plasmid containing gRNA inserts was transferred into *P. tricornutum* via conjugation from *E. coli*. Screening for *Pt2015* knockouts in *P. tricornutum* induced by Cas9 was performed as previously described [8] with minor modifications. After 2–3 weeks, ten *P. tricornutum* exconjugants were randomly selected, inoculated into liquid F/2 medium with 50 μg mL^−1^ zeocin (Invitrogen), and grown for 5 d. The *P. tricornutum* exconjugant cells were lysed and used as the template for polymerase chain reaction (PCR) amplification of the gRNA target site using the specific primers shown in Additional file 1: Table S2. The PCR products were sent for Sanger sequencing to verify the deletion length. Subsequently, liquid cultures of *P. tricornutum* exconjugants that were identified to be edited were diluted to 10^–4^ and plated onto F/2 medium plates containing 50 μg mL^−1^ zeocin and grown for 10–14 d to obtain sub-clones.

### Construction of vectors for Pt2015 overexpression

The coding sequence (444 bp) of the *Pt2015* gene was amplified for overexpression using the primers Pt2015oe-F and Pt2015oe-R, and further digested with *Eco*RI and *Kpn*I, and then inserted into pPha-T1 vector [36]. Additionally, the coding sequence (444 bp) of the *Pt2015* gene for overexpression was amplified with Pt2015eGFP-F and Pt2015eGFP-R, and further digested by *Eco*RI and *Kpn*I, and then inserted into pPHat_eGFP vector. The vectors containing coding sequences of the *Pt2015* gene or the Pt2015-eGFP were introduced into WT *P. tricornutum* using biolistic transformation with the Biolistic PDS-1000/He Particle Delivery System (Bio-Rad, CA, USA) [37], respectively. For the selection of overexpression clones, bombarded cells were plated onto 50% fresh seawater agar plates (1% agar) supplemented with 100 μg mL^−1^ zeocin (Invitrogen). The plates were placed under conditions of 80 μmol photons m^−2^ s^−1^ for 2–3 weeks, and resistant colonies were inoculated into liquid F/2 medium containing 50 μg mL^−1^ zeocin. The transformants were screened using primers (YzF and YzR, Additional file 1: Table S2).

### Cell preparation for electron microscopy

The procedures used for scanning electron microscopy sample preparation were as previously described, with minor modification [30]. The cells were fixed with 5% glutaraldehyde in phosphate buffered saline (PBS, pH 7.4) for 2 h and dehydrated in a series of increasing ethanol concentrations (30%, 50%, 70%, 80%, 90%, and 100%) for 10 min at 4 °C. The cells were treated with isoamyl acetate for 10 min, critical point dried (Hitachi-HCP, Hitachi, Japan), sputter coated with platinum (MC1000, Hitachi), and examined with a scanning electron microscope (S-3400 N, Hitachi).

The ultrastructure of the different samples was examined using transmission electron microscopy as previously described [30]. Cells were fixed with 2.5% glutaraldehyde (Hushi, Shanghai, China) in PBS for 2 h and washed three times for 30 min in 0.1 M PBS at 4 °C. The cells were then post-fixed with 1% osmium tetroxide (Ted Pella, CA, USA) in PBS for 1.5 h and washed three times with 0.1 M PBS at 4 °C. Next, the samples were dehydrated in alcohol (Hushi), infiltrated with acetone (Tieta, Laiyang, China) and an epoxy resin (SPI-CHEM, USA) mixture, embedded, and polymerized in epoxy resin. Ultrathin sections were obtained using a Leica EM UC7 ultramicrotome (Leica Microsystems, Germany) and transferred onto copper grids covered with a Formvar membrane (Electron Microscopy China, Beijing, China). Two-percent uranyl acetate and lead citrate (Ted Pella Inc.) were used for contrast staining. The sections were photographed using a transmission electron microscope (HT7700, Hitachi).

### Fluorescent microscopy

Subcellular localization analysis of chlorophyll autofluorescence and the green fluorescent protein (GFP) fusion of Pt2015 was performed using laser-scanning confocal microscopy (LSM710, Carl Zeiss, 40 ×). Chlorophyll autofluorescence was excited at 633 nm and detected at a bandpass filter of 675–740 nm. GFP fluorescence was excited using an excitation wavelength of 488 nm and detected with a bandpass filter of 510–570 nm.

### Lipid analysis

For lipid body labeling, the cells (1 × 10^6^ cells/mL) of different strains were cultured for 7 d and incubated with BODIPY 505/515 (Thermo Fisher Scientific) at a concentration of 0.5 mg/mL for 7 min in the dark [38]. For cell imaging, BODIPY 505/515 was excited at 488 nm and fluorescence was collected from 510 to 550 nm. Gravimetric means, a conventional lipid quantification method, was used for total lipid analysis. Algal cultures were harvested after cultivation for 7 d by centrifugation at 2500 × *g* for 5 min. Algal cells were frozen in liquid nitrogen and then dried in a freeze dryer. Lipids were extracted from 50 mg dried algal powder using a modified chloroform–methanol system [39]. Crude samples were dried under a N_2_ flow until a constant weight was obtained.

### Plastid purification, membrane solubilization, and immunoblotting

Plastids were purified as previously described [40]. Briefly, cells were centrifuged and re-suspended gently in isolation buffer (0.5 M sorbitol, 50 mM HEPES–KOH, 6 mM ethylenediaminetetraacetic acid (EDTA), 5 mM MgCl_2_, 10 mM KCl, 1 mM MnCl_2_, 1% (w/v) polyvinylpyrrolidone 40, 0.5% bovine serum albumin (BSA), and 0.1% cysteine; pH 7.2–7.5) and slowly passed through a French Press at 90 MPa. The mixture of broken cells was centrifuged at 300 × *g* for 8 min to remove intact cells and cell debris. The supernatant was collected and then centrifuged at 2000 × *g* for 10 min at 4 °C. The pellet containing the plastids was gently re-suspended in washing buffer (0.5 M sorbitol, 30 mM HEPES–KOH, 6 mM EDTA, 5 mM MgCl_2_, 10 mM KCl, 1 mM MnCl_2_, 1% polyvinylpyrrolidone 40, 0.1% BSA; pH 7.2–7.5) and loaded onto a discontinuous Percoll gradient (10%, 20%, and 30%) in the same buffer. After centrifugation (SW40Ti rotor, Beckman Coulter, Indianapolis, IN, USA) at 10,000 × *g* for 35 min, the plastid fraction was recovered in the 20% Percoll layer of the gradient, diluted in washing buffer (without BSA), and subjected to centrifugation at 14,000 × *g* for 10 min at 4 °C. To separate thylakoids and the stroma, plastids were subjected to osmotic shock (induced by incubation for 5 min in the washing buffer without sorbitol), and centrifugation. The supernatants contained the stroma and the pellets comprised the thylakoids, which were re-suspended in washing buffer without sorbitol.

Thylakoid membrane complexes were solubilized and separated as previously described, with minor modification [41]. The thylakoids were solubilized in a buffer (25 mM HEPES–KOH [pH 7.0], 10 mM NaCl, and 5 mM MgCl_2_) containing 1% n-dodecyl-β-D-maltoside (β-DM) with a chlorophyll concentration of 0.5 μg/μL for 10 min on ice. After centrifugation at 15,000 × *g* for 10 min at 4 °C, the supernatant (200 μg chlorophyll) was loaded onto the top of a sucrose density gradient (1.6 M, 1.3 M, 1 M, 0.7 M, 0.4 M, and 0.1 M) containing 25 mM HEPES–KOH (pH 7.0), 10 mM NaCl, 0.02% β-DM, and 5 mM MgCl_2_. Thylakoid protein complexes were separated by ultracentrifugation for 22 h at 220,000 × *g* at 4 °C in a SW40 rotor (Beckman Coulter). Ten fractions were extracted with syringes and equal amounts of each fraction were used for further tricine-sodium dodecyl sulfate-polyacrylamide gel electrophoresis (SDS-PAGE) and immunoblotting analysis.

Immunoblotting analysis was performed as previously described [41]. Antibodies to PsbA, PsbD, PsaA, CytF, Actin, ribulose bisphosphate carboxylase large subunit, and LhcX (LhcSR of moss) were purchased from Agrisera (Vännäs, Sweden). Peptides were designed to match unique sections of Pt2015 (CREWRCKFEGDKSDSE), chemically synthesized, and then injected into rabbits to generate polyclonal antibodies (PhytoAB, San Jose, CA, USA). Horseradish peroxidase-conjugated secondary antibody and an enhanced chemiluminescence detection kit (Tian Gen, Beijing, China) were used for detection.

### Measurement of chlorophyll fluorescence and P700

In vivo chlorophyll *a* fluorescence and P700 oxidation signals were measured simultaneously at 25 °C using a Dual-PAM-100 (Heinz Walz, Germany) in *P. tricornutum* WT, 2015KO, and oeT strains. Before measurements, cells were centrifuged (1500 × *g*, at 20 °C, 3 min) and concentrated 40-fold, and then dark acclimated for 10 min. A saturating pulse of light (300 ms, 10,000 μmol photons m^−2^ s^−1^) was used to determine the maximal fluorescence levels in the dark-adapted state (F_m_) and during actinic light illumination (F_m_′). The steady-state fluorescence level (F_s_) was recorded during actinic light illumination (80 μmol photons m^−2^ s^−1^). The quantum yield [Y(II)] of photosystem II (PSII) was calculated as (F_m_–F_s_)/F_m_′. The Y(NO) and Y(NPQ) were calculated using the Dual-PAM software and saved in a report file. Photosystem I (PSI) was measured in the dual-wavelength mode (photodetector set to measure 875 nm and 830 nm pulse-modulated light). The maximum P700 signal, P_m_, was determined by the application of the saturation pulse in the presence of far-red light. The P700 signal, P, was determined just before the saturation pulse. P_m_′ is the maximum P700 signal induced by combined actinic illumination with the saturation flash. Y(I) was calculated as (P_m_′–P)/P_m_. The acceptor-side limitation of PSI (Y(NA)) was calculated as (P_m_–P_m_′)/P_m_, while the donor-side limitation of PSI (Y(ND)) was calculated as 1–Y(I)–Y(NA)[42, 43].

### Measurements of photosynthetic O_2_ evolution

Rates of photosynthetic O_2_ evolution of the WT, 2015KO, and oeT strains under different light conditions and dark respiration were measured using a Clark-type oxygen electrode (Hansatech, Norfolk, UK). Cells were concentrated via centrifugation at 1500 × *g* for 3 min. After centrifugation, cells were re-suspended in fresh F/2 culture medium. The OD_730_ value was adjusted to 1, and 2 mL was placed into the electron chamber. Before measurement under different light conditions, the samples were dark acclimated for 10 min. After the dark pre-treatment, the rate of O_2_ evolution in the chamber was measured over a range of light intensity (50–2800 μmol photons m^−2^ s^−1^), provided by an LED light source peaking at 630–640 nm. In addition, after the dark pre-treatment, the rate of O_2_ evolution of the cells was measured under a light intensity of 100 and 2000 μmol photons m^−2^ s^−1^, respectively.

### Cultivation of amoebae

The amoebae used in this study belongs to the Heterolobosea class which was isolated from large-scale cultivation of *P. tricornutum*. The spores of amoebae were centrifugated at 3500 × *g* for 5 min, and then diluted with WT and oeT cells with the same cell density (5 × 10^6^ cells/ml), respectively. The diluent was cultured in culture plates (6 wells) at 25 °C under the dark condition. After 24 h, the cells concentrations of the WT and oeT were counted.

### Salinity and temperature experiments of the oeT strain

For the salinity experiments, three salinity conditions (100%, 50%, and 30% seawater) were tested using F/2 medium. Nutrient trace metals and vitamins were the same as in the F/2 medium, except the salts were diluted to 50% or 30% seawater. The oeT strain, which was cultured in artificial seawater (100% seawater) for at least 2 months, was moved to three salinity conditions (100% (control), 50%, and 30%) at a starting density of 1 × 10^6^ cell mL^−1^, followed by weekly cell counting for 60 d. All three salinity treatments were performed at 20 °C. Meanwhile, two temperature conditions (20 °C and 10 °C) with the same salinity (100% seawater) were tested at a starting density of 1 × 10^6^ cell mL^−1^, followed by cell counting weekly for 60 d. During this period, the F/2 medium was supplemented every 3 weeks and the percentage of abundance of different cell morphologies (triradiate and fusiform) was quantified by counting in a counting chamber.

### RNA extraction, sequencing, and bioinformatics analysis

Total RNA was extracted using the TRIzol reagent kit (Invitrogen), according to the manufacturer’s protocol, and was assessed using an Agilent 2100 Bioanalyzer (Agilent Technologies, Palo Alto, CA, USA) and electrophoresis. The enriched mRNA was fragmented into short fragments and reverse transcribed into cDNA. The cDNA fragments were purified, end repaired, and a poly(A) tail was added. Then the fragments were ligated to Illumina sequencing adapters. The ligation products were size selected using electrophoresis, PCR-amplified, and sequenced using an Illumina Novaseq 6000 by Gene Denovo Biotechnology Co. Ltd. (Guangzhou, China).

To obtain high quality clean reads, reads were further filtered using FASTP (version 0.18.0) (version 0.18.0) [44]. Ribosome RNA reads were removed after alignment with Bowtie2 (version 2.2.8) [45]. The remaining clean reads were assembled and mapped to the *P. tricornutum* genome (National Center for Biotechnology Information—Assembly: ASM15095v2) using HISAT2 2.4 under default parameters [46]. The mapped reads of each sample were assembled using StringTie v1.3.1 [47]. For each transcription region, a fragment per kilobase of transcript per million mapped reads value was calculated to quantify its expression abundance and variation. Principal component analysis was performed using R package gmodels (http://www.r-project.org/), which is a statistical procedure that converts hundreds of thousands of correlated variables (gene expression) into a set of values of linearly uncorrelated variables called principal components. Differential expression analysis of RNA was performed using DESeq2 software between two different groups (false discovery rate (FDR) < 0.05, absolute fold change ≥ 2) [48]. Gene function classification was annotated using Gene Ontology (GO) and KEGG enrichment (FDR ≤ 0.05) [49].

### Tandem Mass Tag (TMT)-based quantitative proteomic analysis

Proteomic analysis of the samples (WT, 2015KO, and oeT strains) was performed by PTM-Bio labs Co. Ltd. (Hangzhou, Zhejiang, China). The samples were ground in liquid nitrogen, and the powder was transferred to a 5 mL centrifuge tube and then sonicated in a lysis buffer (8 M urea, 1% Triton X-100, 10 mM dithiothreitol, 1% protease inhibitor cocktail, 3 μM Trichostatin A, 50 mM N-(9-acridinyl)maleimide, and 2 mM EDTA). The precipitate was then reconstituted with 8 M urea, and the protein concentration was measured using a bicinchoninic acid kit. For trypsin digestion, the protein solution was reduced with 5 mM dithiothreitol at 56 °C for 30 min and alkylated with 11 mM iodoacetamide at room temperature for 15 min in the dark.

After trypsin digestion, the peptides were desalted using a Strata X C18 PSE column (Phenomenex, USA) and freeze dried under vacuum. For TMT labeling, the samples were reconstituted in 0.5 M triethylammonium bicarbonate and processed according to the TMT kit instructions (Thermo-Scientific). The detailed procedures were performed in accordance with previous methods [50, 51].

For high-performance liquid chromatography fractionation, the peptides were fractionated using high-pH reverse-phase high-performance liquid chromatography with an Agilent 300Extend C18 column (Agilent, Santa Clara, CA, USA) (5 mm particles, 4.6 mm internal diameter). The peptides were first separated into 80 fractions over 80 min using a gradient of 2%–60% acetonitrile (pH 9), after which the peptides were combined into 18 fractions and dried by vacuum centrifugation. The peptides were then subjected to a nanospray ionization source followed by tandem mass spectrometry with Q Exactive™ Plus (Thermo-Scientific) coupled online to ultra-performance liquid chromatography.

The tandem mass spectrometry data were processed using the Maxquant search engine (v.1.5.2.8). Tandem mass spectra were searched against the *P. tricornutum* UniProt database combined with a reverse decoy database. Trypsin/P was specified as a cleavage enzyme, allowing up to two missing cleavages. The mass tolerance for precursor ions was set at 20 ppm in the first search and 5 ppm in the main search. The mass error was set to 0.02 Da for the precursor ions and fragment ions. Carbamidomethylation on cysteine was specified as a fixed modification and oxidation on methionine was specified as a variable modification. FDR thresholds for protein, peptide, and modification site identification were specified at 1%. TMT-6-plex was selected as the quantification method. All other parameters in MaxQuant were set to default values.

The functional annotation of the differentially expressed proteins (DEPs) was annotated to the GO and Kyoto Encyclopedia of Genes and Genomes (KEGG) databases. The DEP IDs were converted to UniProt IDs, and then the UniProt IDs were used to match the GO ID, and the corresponding information was retrieved from the UniProt-GOA database (http://www.ebi.ac.uk/GOA/) according to the GO ID. Un-annotated proteins were annotated by InterProScan using the protein sequence alignment method. All annotated proteins were then classified into three categories: biological process (BP), cellular component (CC), and molecular function (MF). The KEGG database (http://www.genome.jp/kegg/) was used to annotate the protein pathway.

### Statistical analysis

Data are expressed as the mean value of four independent experiments (± standard deviation). Data were analyzed using analysis of variance with the SPSS 13.0 statistical software (IBM, Armonk, NY, USA). An independent-samples *t*-test was used at the α = 0.05 significance level to determine whether significant differences existed between the WT, 2015KO, and oeT strains.

## Results

### Bioinformatics analysis of *Pt2015*

*Pt2015* (ID: 7202015, PHATRDRAFT_47103) is localized in chromosome 12 of *P. tricornutum*, and contains one exon and no introns. It encodes a protein consisting of 147 amino acids with a predicated molecular mass of 16 kDa (Additional file 1: Fig. S1A). The program SignalP 4.1 predicted that the cleavage site of the signal peptide in Pt2015 was between the 17th and 18th amino acid (Additional file 1: Fig. S1B). In addition, the TMHMM Server 2.0 predicted that Pt2015 does not possess a transmembrane domain (Additional file 1: Fig. S2). Phylogenetic analysis demonstrated that highly homologous proteins were only found in marine algae, mainly including diatoms, chrysophytes, dinoflagellates, and other red-tide algae, and not in freshwater algae (*Chlamydomonas*) or higher plants (*Arabidopsis*) (Fig. 1). A comparison of the conserved regions in Pt2015 proteins from diatoms, chrysophytes, dinoflagellates, and other red-tide algae revealed that five conserved motifs were identified in Pt2015. In particular, the two C-terminal motifs were much more conserved in Pt2015 (Additional file 1: Fig. S3A). Interestingly, Pt2015 was highly homologous with part of the sequence of exosome component 10 in the unicellular red algae *Porphyridium purpureum* (Additional file 1: Fig. S3B).

### Subcellular localization of Pt2015 protein

**Fig. 2:**
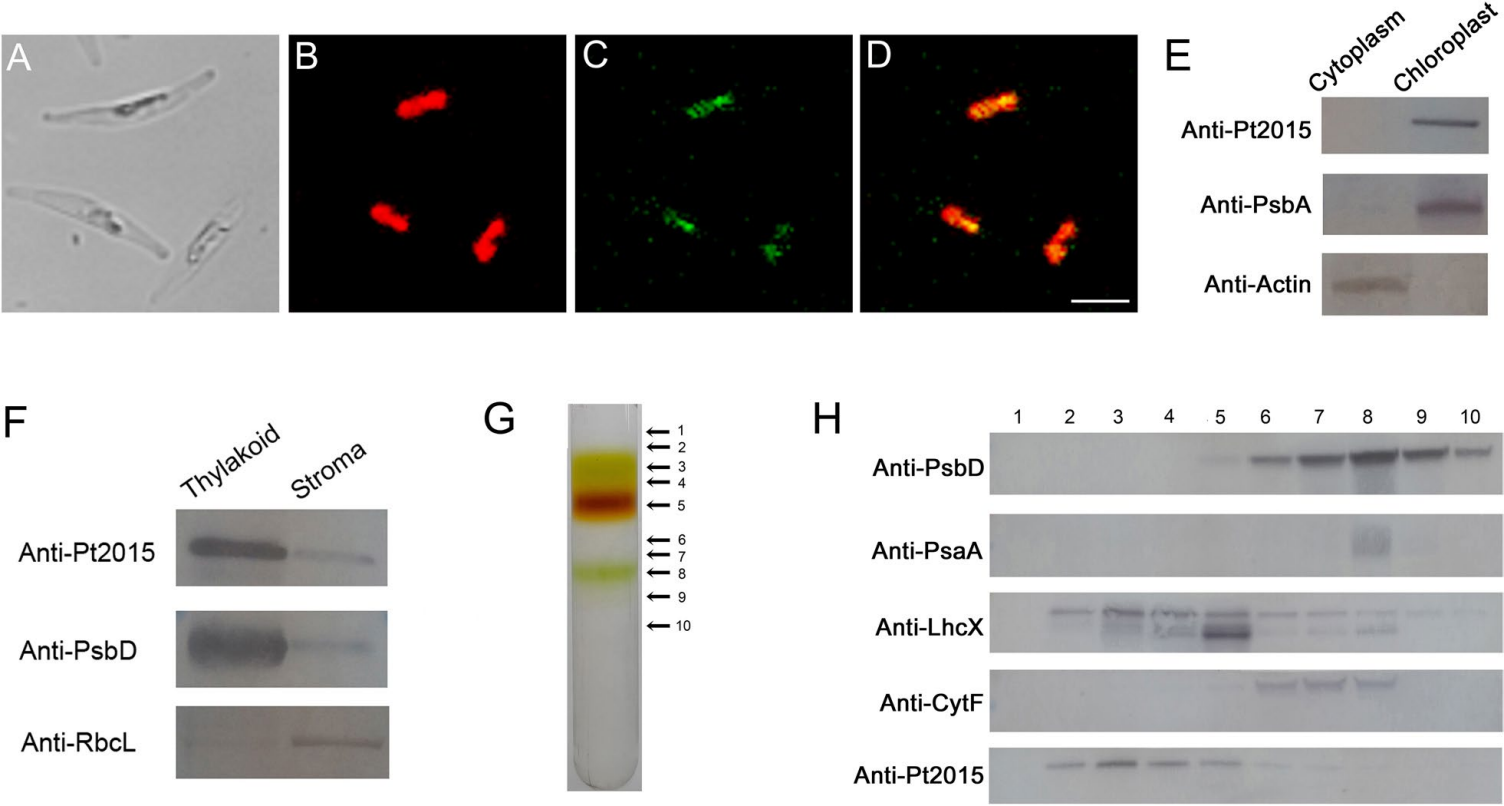
Subcellular localization of Pt2015. Fluorescent microscopy of Pt2015 green fluorescence protein (GFP) fusion proteins in a *P. tricornutum* Pt2015:GFP mutant. **A** Light image, **B** Chlorophyll autofluorescence, **C** GFP fluorescence, **D** Merged image of (**B** and **C**) (Scale bar, 5 μm). **E** Immunoblotting analysis of Pt2015. The cytoplasm and plastid were extracted from the wild-type (WT) strain. **F** Immunoblotting analysis of Pt2015. Plastids were extracted from the WT strain and then fractionated into thylakoid membranes and stroma. **G** The thylakoid membranes have been solubilized using n-dodecyl-β-D-maltoside (β-DM) and subjected to sucrose density gradient ultracentrifugation. The solubilized thylakoid membranes (200 μg chlorophyll) have been loaded on a gradient. The gradient was divided into ten fractions according to band color. **H** An equal volume of each fraction was used for immunoblotting analysis using antibodies against PsbD, PsaA, CytF, LhcSR, and Pt2015, respectively

Subcellular localization of Pt2015 was performed by expressing a C-terminal GFP fusion protein in *P. tricornutum*. The fluorescence signal associated with the Pt2015:GFP transformant was visible within the plastid (Fig. 2A–D). Additionally, intact plastids were further purified from the *P. tricornutum* WT strain through a discontinuous Percoll gradient (Additional file 1: Fig. S4). As shown in Fig. 2E, immunoblotting analysis demonstrated that Pt2015 was observed in the plastid, with a localization similar to that of PsbA (a protein located on thylakoids), but different to that of actin (a cytoplasm marker). This finding was consistent with an in silico analysis of the signal peptide sequence in Pt2015 (Additional file 1: Fig. S1). Therefore, it was concluded that Pt2015 resides in the plastid.

Interestingly, although there was no transmembrane domain in Pt2015 (Additional file 1: Fig. S2), immunoblotting results indicated that Pt2015 was located on the thylakoids, with a localization similar to that of PsbD (a protein located on thylakoids), although small signals of both proteins were also detected in the stroma fraction which might be caused by the cross-contamination of the stroma (Fig. 2F). These results suggested that Pt2015 is a peripheral protein of thylakoids in diatoms. Subsequently, thylakoids from the *P. tricornutum* WT were solubilized with a mild detergent (β-DM), loaded onto a sucrose density gradient, and ultracentrifuged. After centrifugation, the gradient from top to bottom was divided into 10 fractions (10 bands, 1 to 10) according to band color (Fig. 2G). To identify polypeptide composition, each band was analyzed using tricine SDS-PAGE and immunoblotting analysis (Fig. 2H). PSII was detectable in bands 7, 8, and 9, while PSI was mainly present in band 8. LhcX was detected in bands 3, 4, and 5, and LhcX in band 3 was significantly different from that of band 5. It should be noted that the immunoblotting analysis of LhcX identified antibodies against LhcSR of *Physcomitrella patens*. Pt2015 signals were mainly detected in bands 2–5, especially in band 3. These results were in agreement with the analysis of the two dimensional Blue Native/SDS-PAGE (Additional file 1: Fig. S5).

### Impact of *Pt2015* knockout and overexpression on cell morphotype and photosynthesis

**Fig. 3:**
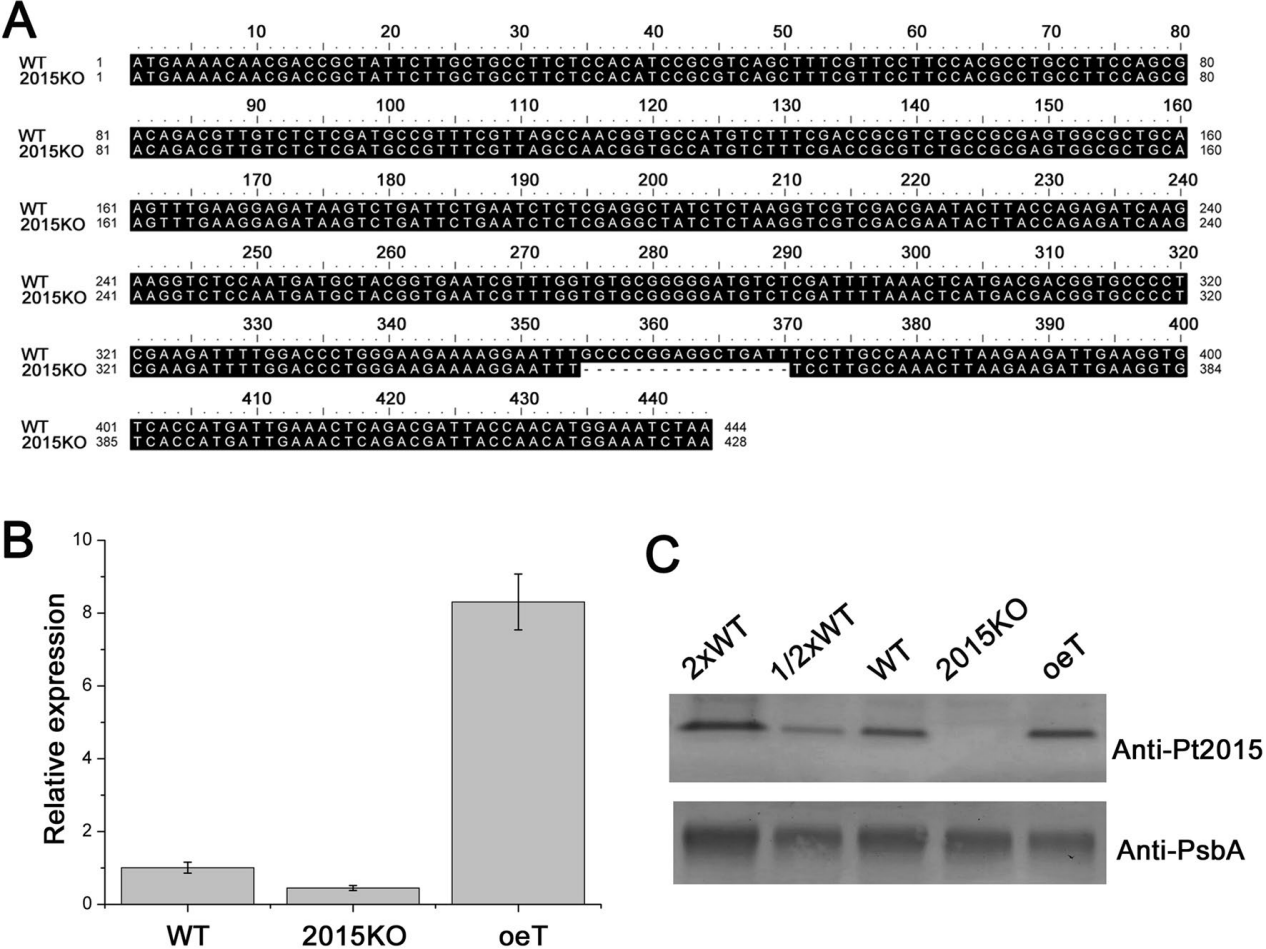
Characterization of the *Pt2015* knockout strain (2015KO). **A** Characteristics of the 2015KO. Sequences of the 2015KO strain and wild type (WT) were aligned. The mutation in the *Pt2015* locus compared to the WT sequence was indicated, in which 16 bp are deleted. **B** Quantitative reverse transcription polymerase chain reaction (qRT–PCR) analysis of *Pt2015* mRNA levels in the WT, 2015KO, and the *Pt2015* overexpression (oeT) strains. Ribosomal protein S (*RPS*) was used as reference gene. Error bars represent the standard deviation (n = 3). **C** Immunoblotting analysis of the Pt2015 protein in the WT, 2015KO, and oeT strains. Photosystem II protein D1 (PsbA) protein level was detected as the loading control. Fifteen micrograms protein extracts of the WT, 2015KO, and oeT strains have been loaded

Using the CRISPR/Cas9 method, a *Pt2015* knockout line (named 2015KO) was obtained in which the Pt2015 protein was thoroughly knocked out. As shown in Fig. 3A, in the 2015KO strain, 16 bases were deleted between bases 355 and 370, which is the gRNA3 binding site. In addition, a Pt2015 overexpression strain (oeT) was obtained. Both quantitative reverse transcription polymerase chain reaction (qRT–PCR) and immunoblotting analysis suggested that Pt2015 was knocked out in 2015KO and overexpressed in the oeT strains, respectively (Fig. 3B and C).

**Fig. 4:**
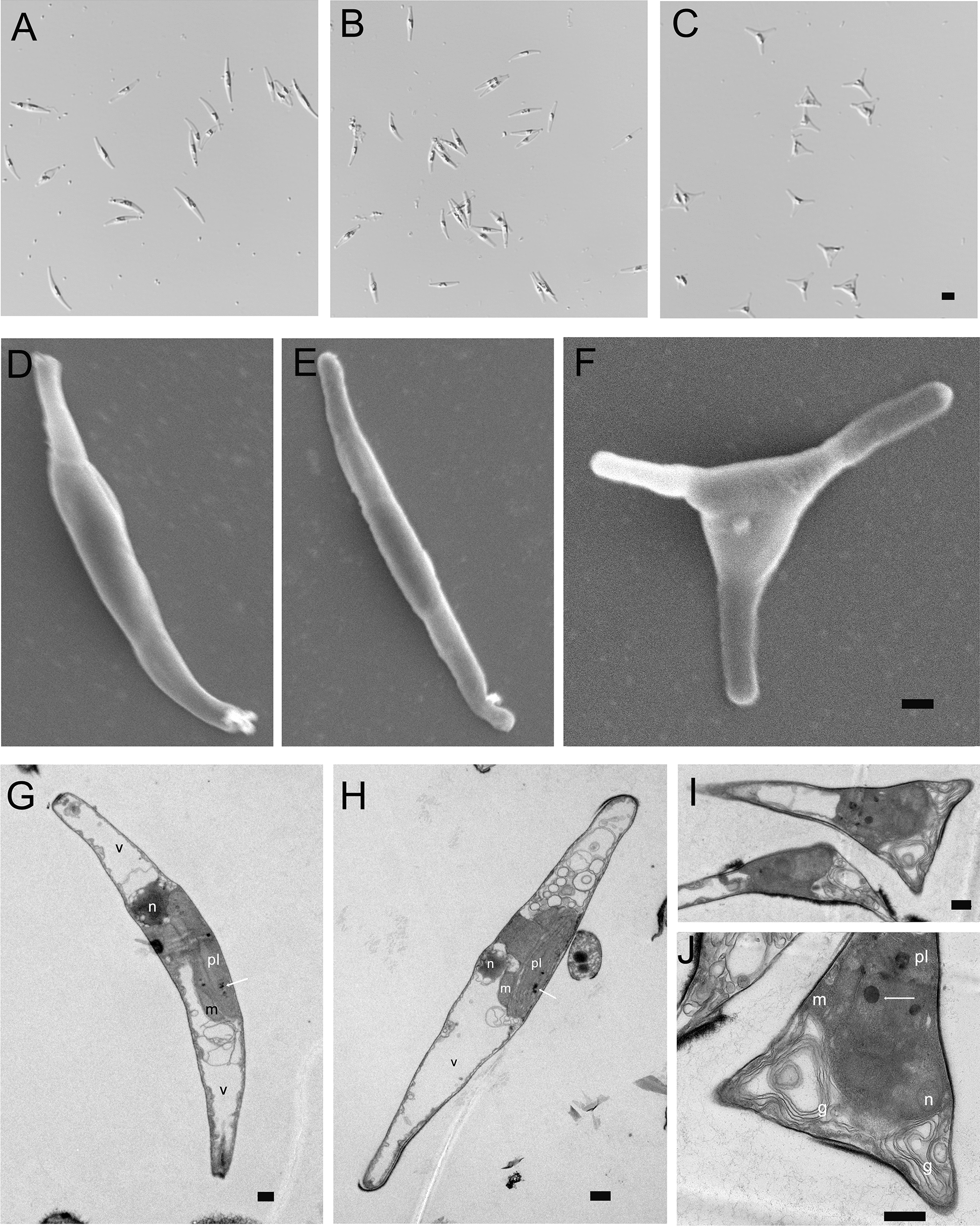
Micrographs of the wild type (WT), *Pt2015* knockout strain (2015KO), and the *Pt2015* overexpression (oeT) strains. Micrographs of the WT (**A**), 2015KO (**B**), and oeT (**C**) strains imaged using differential interference contrast (DIC) optics (all DIC images are shown at the same scale and the scale bar: 5 μm). Scanning electron microscopy (SEM) micrographs of the WT (**D**), 2015KO (**E**), and oeT (**F**) strains. The scale bar is 0.5 μm. Transmission electron microscopy (TEM) micrographs of the WT (**G**), 2015KO (**H**), and oeT (**I** and **J**) strains. White arrows indicate lipid droplets. The scale bar is 0.5 μm

Interestingly, the oeT strain displayed a triradiate morphotype, in which cells were 95% triradiate (the rest were fusiform) (Fig. 4A–C). In contrast, the 2015KO strain demonstrated a fusiform morphotype, which was similar to the WT. Furthermore, the WT, 2015KO, and oeT strains were observed using scanning electron microscopy (Fig. 4D–F). These data suggested that there was no difference in the size and shape of fusiform cells between the 2015KO and WT strains (Fig. 4D and E). In contrast, the triradiate cells in the oeT strain demonstrated nonsymmetrical and unequal arm lengths (Fig. 4F). In addition, the ultrastructure of the WT, 2015KO, and oeT strains was analyzed using TEM (Fig. 4G–I, and J). These data suggested that similar organelles were found in the WT, 2015KO, and oeT strains, including nuclei, plastids, mitochondria, and vacuoles. A large plastid was localized near the nucleus in the WT, 2015KO, and oeT strains. Vacuoles occupied the distal arms of the three strains, which were larger in the WT and 2015KO strains. Mitochondria were observed close to the plastid in the three strains. Furthermore, considerably more Golgi apparatus were observed in the oeT strain (Fig. 4I and J). In addition, lipid droplets in the oeT strain were larger than those in the WT and 2015KO strains (Fig. 4I and J).

**Fig. 5:**
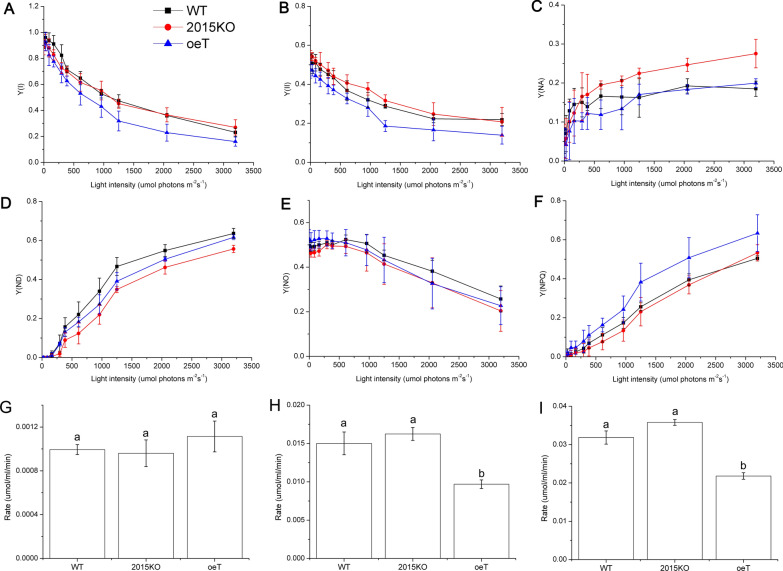
Impact of *Pt2015* knockout and overexpression on photosynthetic activity. The light intensity dependence of photosynthetic parameters has been monitored in the wild type (WT), *Pt2015* knockout strain (2015KO), and the *Pt2015* overexpression (oeT) strains. The quantum yield of photosystem I [Y(I)] (**A**), the quantum yield of photosystem II [Y(II)] (**B**), Y (NA) (**C**),Y (ND) (**D**), Y (NO) (**E**) and Y (NPQ) (**F**). **G** The rate of oxygen evolution in the WT, 2015KO, and oeT strains under 100 μmol photons m^−2^ s^−1^. **H** The rate of oxygen evolution in the WT, 2015KO, and oeT strains under 2000 μmol photons m^−2^ s^−1^. **I** The rate of oxygen evolution in the WT, 2015KO, and oeT strains under increasing light intensity (50, 100, 200, 400, 800, 1600, and 2800 μmol photons m^−2^ s^−1^). Data represent the mean ± standard deviation (SD) of four biological replicates. The different letters indicate that there was significant difference between WT, 2015KO, and oeT

The photosynthetic electron transport of the WT, 2015KO, and oeT strains was analyzed using a Dual-PAM system (Fig. 5). The Y(II) was lower in the oeT strain compared to the WT and 2015KO strains (Fig. 5A). Y(I) displayed a similar trend as the Y(II) (Fig. 5B). Under high light conditions, the Y(NA) value in the oeT strain was much higher than that of the WT and 2015KO strains, suggesting that there was a limitation in electron acceptance from PSI, especially under high light conditions (Fig. 5C). Moreover, there were no significant differences between Y(ND) and Y(NO) among the oeT, 2015KO, and WT strains (Fig. 5D and E). Nevertheless, the Y(NPQ) in the oeT strain was higher than that in the WT and 2015KO strains (Fig. 5F). Additionally, the photosynthetic oxygen rate of the WT, 2015KO, and oeT strains was analyzed (Fig. 5G–I). The photosynthetic oxygen evolution rate of the oeT strain, particularly under higher light (μmol photons m^−2^ s^−1^) conditions (Fig. 5H), was much lower than that of the WT and 2015KO strains (Fig. 5G and H). In contrast, the oxygen evolution rate of the 2015KO strain was even higher than that of the WT strain. This was consistent with the Y(II) data using the Dual-PAM.

### The oeT strain possessed a higher lipid content, demonstrated grazing resistance, and was insensitive to hyposalinity and low temperature

**Fig. 6:**
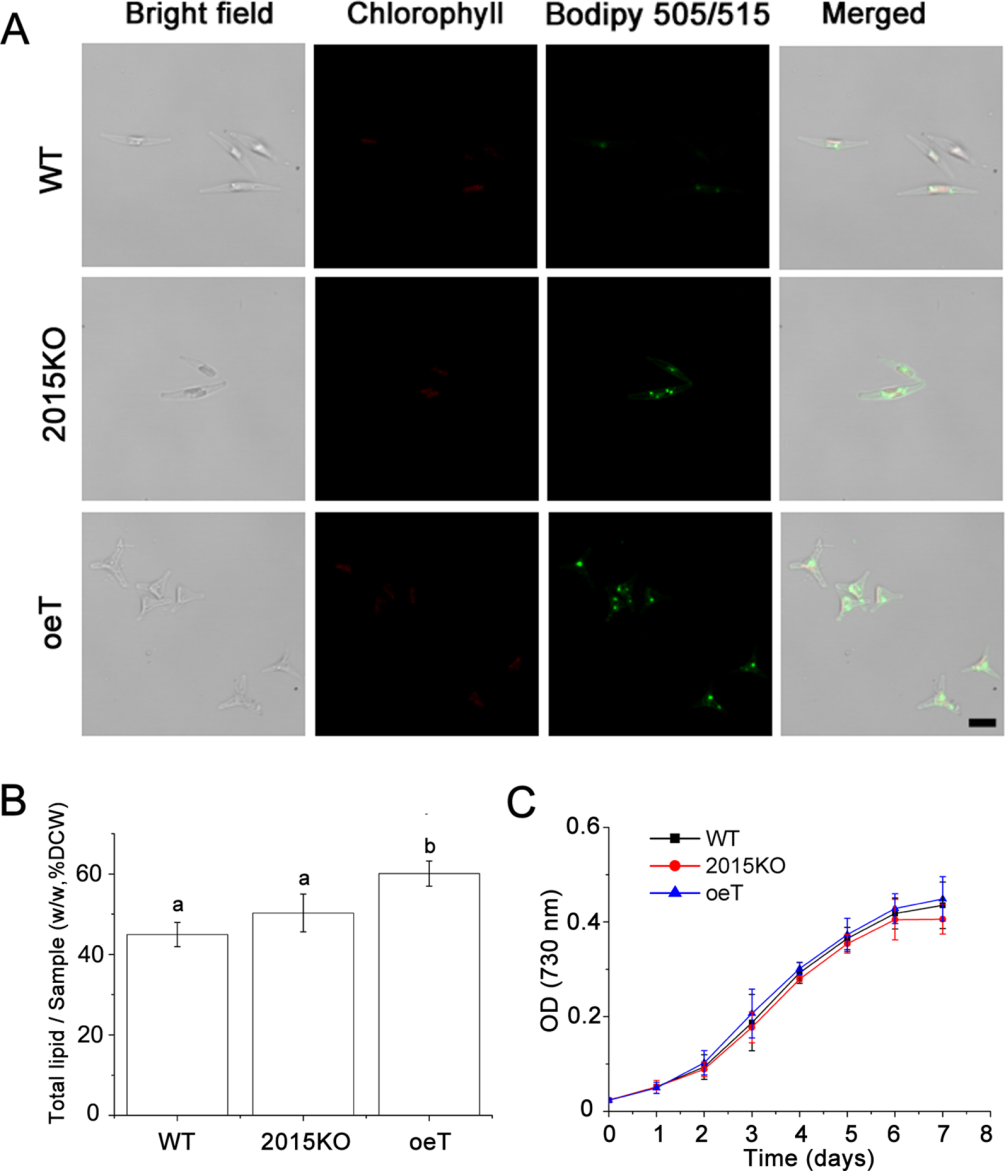
Lipids in the wild type (WT), *Pt2015* knockout strain (2015KO), and the *Pt2015* overexpression (oeT) strains. **A** Localization of lipid bodies in the WT, 2015KO, and oeT strains after staining with BODIPY 505/515. Bright-field micrographs, plastid autofluorescence, BODIPY 505/515 images, and a combination of BODIPY 505/515 images (merged) with plastid autofluorescence are shown. All images are shown at the same scale. The scale bar is 5 μm. **B** Total lipid content of WT, 2015KO, and oeT strains using gravimetric means. Different letters represent significant differences of total lipid content in the WT, 2015KO, and oeT strains. **C** Growth curves of WT, 2015KO, and oeT strains at 20 °C under 80 μmol photons m^−2^ s^−1^

**Fig. 7:**
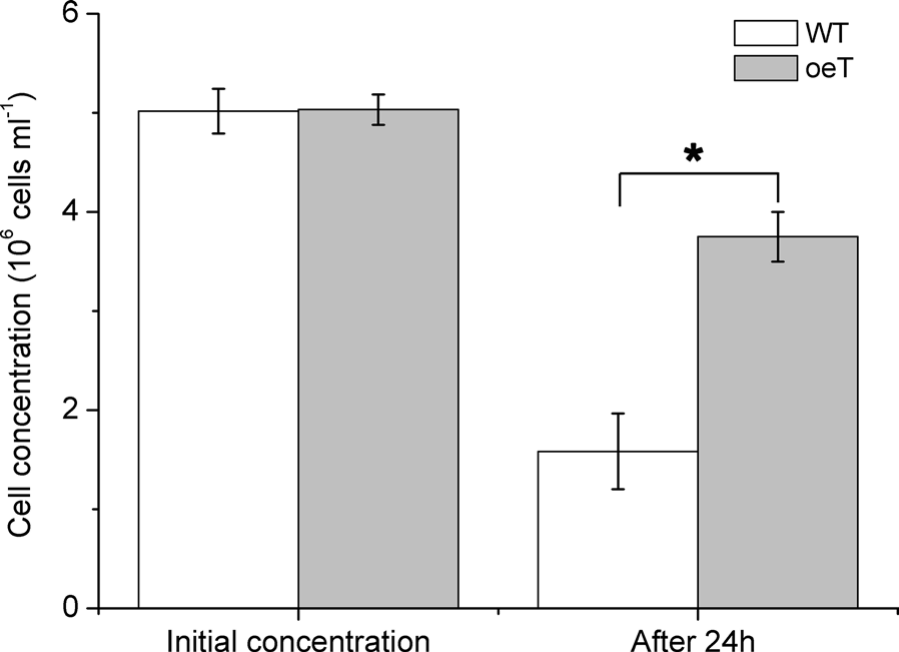
Changes of cells concentrations of the oeT and WT strains during the cultivation with amoebae. The spores of amoebae were centrifugated at 3500 × *g* for 5 min, and then the precipitation was diluted with WT and oeT cells at the same cell density (5 × 10^6^ cells/ml), respectively. The diluent was cultured in culture plates (6 wells) at 25 °C under the dark condition. After 24 h, the cells of the oeT and WT were counted. The asterisk indicates that there was significant difference between WT and oeT

The lipid content in the oeT strain was clearly different from that in the WT and 2015KO strains. Spherical lipid bodies (also called lipid droplets) were observed in cells of the WT, 2015KO, and oeT strains labeled with BODIPY 505/515 and were generally distributed close to the plastid (Fig. 6). In the oeT cells, lipid bodies were also observed in the distal arm. Additionally, the oeT cells contained larger and more lipid bodies than WT and 2015KO. Total lipid content in the WT, 2015KO, and oeT strains was further analyzed using gravimetric means. These data showed that total lipid content in the oeT strain was higher than that in the WT and 2015KO strains, which increased by approximately 30% compared to that of the WT strain (Fig. 6B). This was consistent with observations of BODIPY 505/515-labeled cells and the transmission electron microscopy results. Although the morphotypes and lipid content of WT, 2015KO, and oeT changed significantly, the growth characteristics among the WT, 2015KO, and oeT strains did not demonstrate significant differences (Fig. 6C). In addition, to test the resistance of the oeT to microzooplankton, the amoebae (the Heterolobosea class) which was isolated from large-scale cultivation of *P. tricornutum* was used in this study. It should be noted that the 2015KO strain demonstrated a fusiform morphotype, which was similar to the WT, so in the grazer test the fusiform was used as control. The results suggested that during the co-culture of *P. tricornutum* and amoebae, after 24 h the cell concentrations of oeT strain were significantly higher than that of WT (Fig. 7 and Additional file 1: Fig. S12).

It has been reported that the triradiate strain isolated from a natural condition was very sensitive to low salinities and low temperatures [30]. Therefore, to test the effects of hyposalinity and low temperatures on morphotypes of the oeT strain, four different experimental conditions were used: 100% seawater (normal seawater) and 20 °C, 50% seawater and 20 °C, 30% seawater and 20 °C, and 100% seawater and 10 °C. The condition comprising 100% seawater and 20 °C was the control (normal condition). Under the condition of 0% seawater, the oeT cells bleached and eventually died; therefore, this condition was not analyzed in this study. Additionally, during treatment under the above-mentioned conditions, fresh culture medium was supplemented every 3 weeks. As shown in Additional file 1: Fig. S6, under the two hyposalinity conditions (50% and 30% seawater) and long-term culture (60 d), the abundance of fusiform and triradiate morphotypes in the oeT strain did not clearly change and abundance was maintained at 5% and 95%, respectively (Additional file 1: Fig.S6B and C). The trends in these two conditions were like that under normal conditions (Additional file 1: Fig. S6A). Moreover, under low temperature conditions (100% seawater and 10 °C) and long-term culture (60 d), the abundance of fusiform and triradiate morphotypes in the oeT strain was also maintained at a constant level (5% and 95%, respectively) (Additional file 1: Fig. S6D). These data suggested that the oeT strain (triradiate cells) was not sensitive to hyposalinity and low temperature.

### Transcriptomic and proteomic analysis of WT, 2015KO, and oeT

**Fig. 8:**
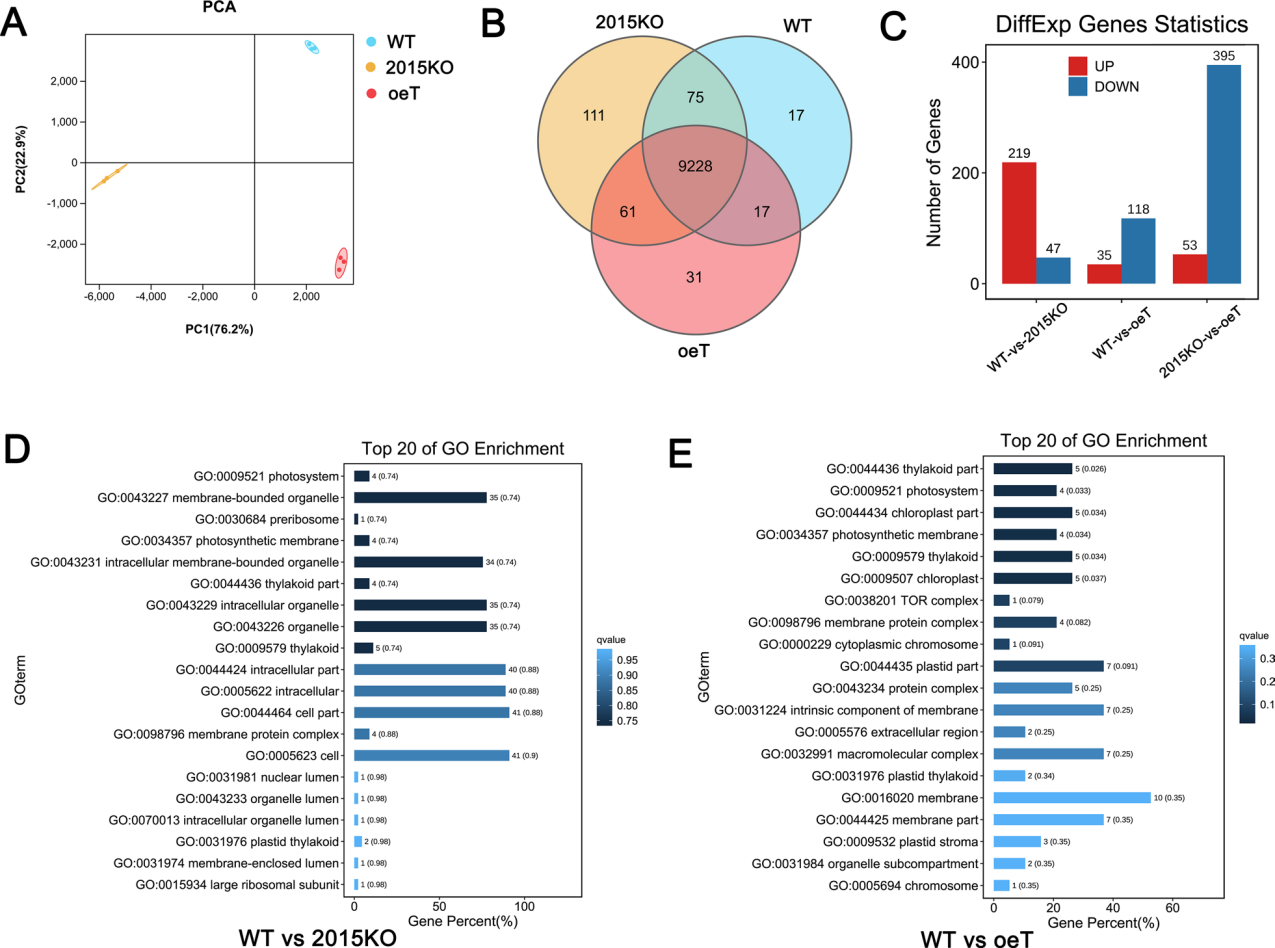
Transcriptomic analysis of the wild type (WT), *Pt2015* knockout strain (2015KO), and *Pt2015* overexpression (oeT) strains. **A** Principal components analysis (PCA) of gene expression in the WT, 2015KO, and oeT strains. **B** Venn diagram of gene expression in the WT, 2015KO, and oeT strains (FPKM > 2). **C** Pairwise comparison of differently expressed genes (up and downregulated genes) in the WT, 2015KO, and oeT strains. Bar graph showing up- or downregulated gene counts (fold change > 2, false discovery rate [FDR] < 0.05). **D** Gene ontology (GO) component enrichment analysis of the pairwise comparison between the WT and 2015KO strains. **E** GO component enrichment analysis of the pairwise comparison between the WT and oeT strains

To gain insight into the gene expression profiles associated with the WT, 2015KO, and oeT strains, the mRNA transcriptome of these three strains was explored using high-throughput RNA-Sequencing (RNA-Seq). Validation of the RNA-Seq data was conducted using qRT–PCR experiments analyzing the expression of eight genes selected randomly from the list of differently expressed genes (DEG). qRT–PCR data suggested the expression of the selected genes was consistent with the RNA-Seq data, suggesting the transcriptomic data were of high quality (Additional file 1: Fig. S7). Principal component analysis suggested that either *Pt2015* knockout or overexpression greatly affected gene expression (Fig. 8A). Furthermore, pairwise comparison of gene expression data of the WT, 2015KO, and oeT strains was performed (Fig. 8B and C). Pairwise comparison between the oeT and WT strains suggested that 153 genes were significantly differentially expressed between the two morphotypes, in which 35 genes were upregulated and 118 genes were downregulated. When comparing the oeT and 2015KO strains, 53 genes were upregulated and 395 genes were downregulated.

To characterize the biological process (BP), cellular component (CC), and molecular function (MF) in which the DEGs were involved, a GO analysis was performed. The bar chart of the WT-vs-2015KO and WT-vs-oeT comparisons suggested that many CCs (Fig. 8D and E), BPs (Additional file 1: Fig. S8A and B), and MFs (Additional file 1: Fig. S8C and D) were regulated. The CCs mainly included the plastid part involving the photosynthetic membrane (Fig. 8D and E). Among the BPs in the WT-vs-oeT comparison, the single-organism metabolic process (GO: 0,044,710), DNA metabolic process (GO: 0,006,259), and protein modification process (GO: 0,006,464) were regulated (Additional file 1: Fig. S8B). In addition, similar results were obtained when considering the MF of the WT-vs-oeT comparison, which mainly included molecular transducer activity, tetrapyrrole binding, and oxidoreductase activity (Additional file 1: Fig. S8D). Furthermore, KEGG enrichment analysis of the WT-vs-oeT comparison suggested that nitrogen metabolism and many signaling pathways were regulated (Additional file 1: Fig. S9B). These were also observed in the comparison between the WT and 2015KO strains (Additional file 1: Fig.S8A, S8C and S9A). These data suggested that both the knockout and overexpression of the Pt2015 protein affected the above-mentioned BPs.

**Fig. 9:**
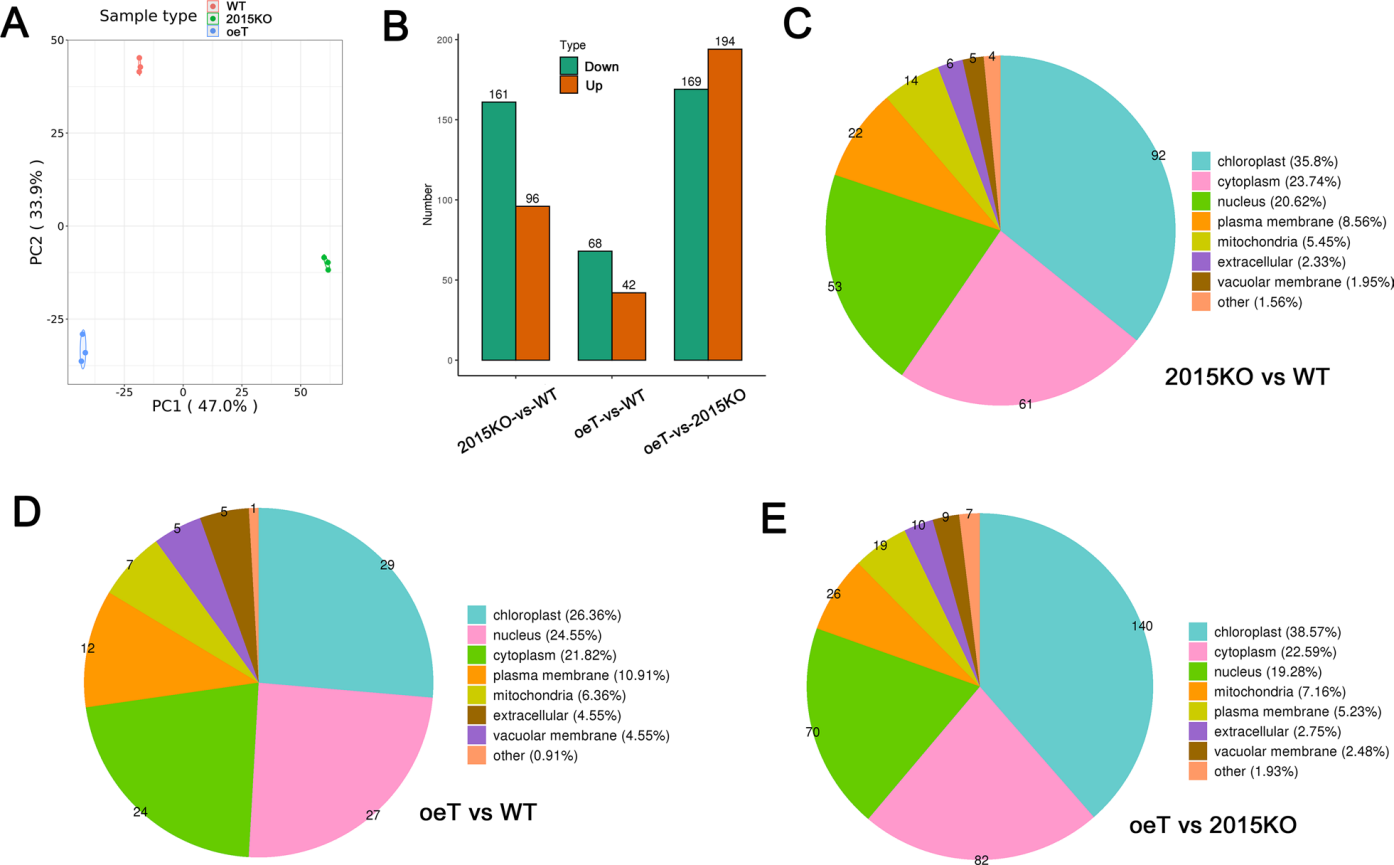
Quantitative proteomic analysis of the wild type (WT), *Pt2015* knockout strain (2015KO), and *Pt2015* overexpression (oeT) strains. **A** Principal components analysis (PCA) of the protein composition in the WT, 2015KO, and oeT strains. **B** Pairwise comparison of differentially expressed proteins (up- and downregulated) in the WT, 2015KO, and oeT strains. **C** Functional classification analysis between the 2015KO and WT strains. **D** Functional classification analysis between the oeT and WT strains. **E** Functional classification analysis between the oeT and 2015KO strains

**Fig. 10:**
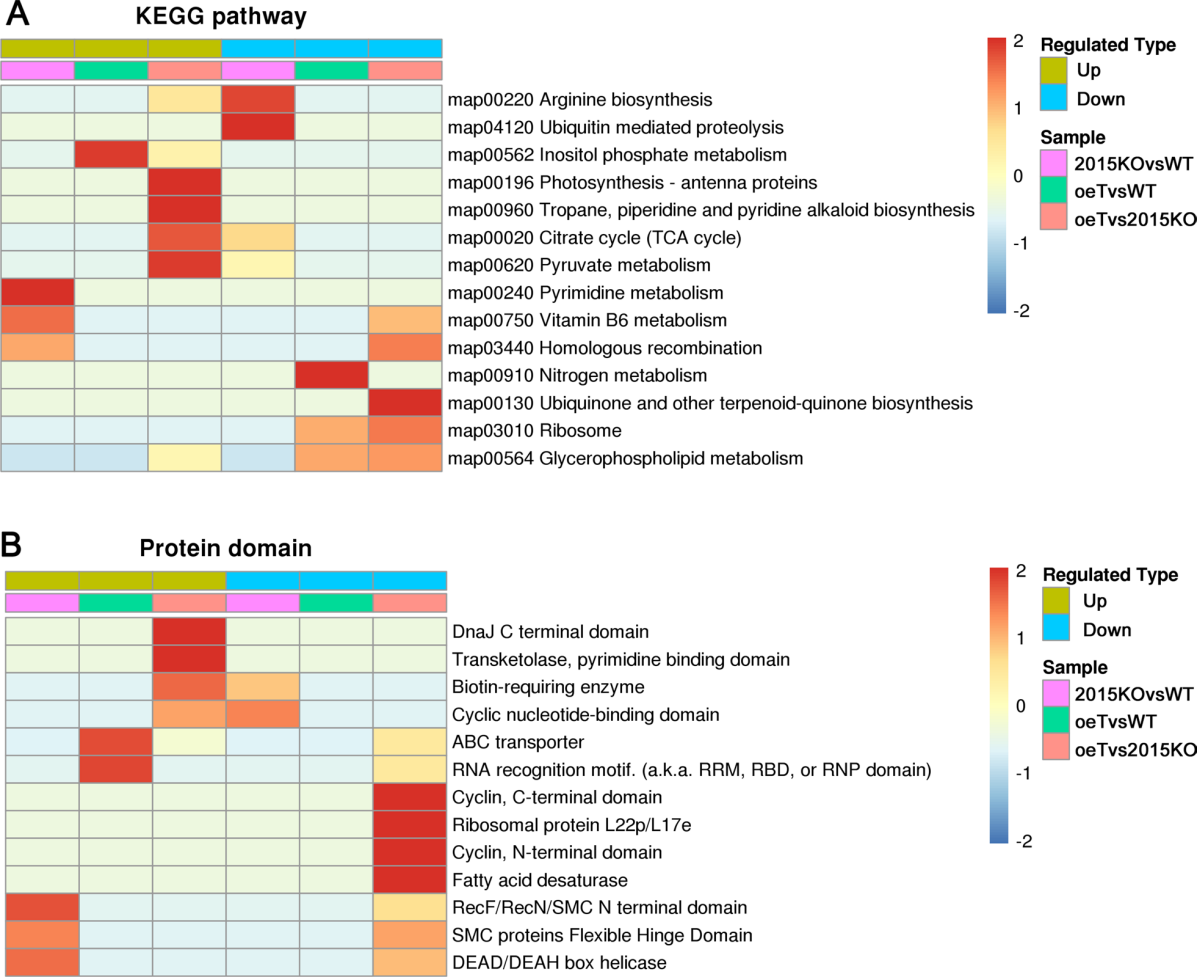
Functional enrichment analysis of differentially expressed proteins (up- and downregulated) in the wild type (WT), *Pt2015* knockout strain (2015KO), and *Pt2015* overexpression (oeT) strains. Kyoto Encyclopedia of Genes and Genomes (KEGG) pathway analysis (**A**) and protein domain analysis (**B**) of differentially expressed proteins in the WT, 2015KO, and oeT strains

To understand the protein levels associated with the WT, 2015KO, and oeT strains, quantitative proteomics were performed. These data suggested that the three strains (WT, 2015KO, and oeT) demonstrated different protein compositions (Fig. 9A). Moreover, 6680 proteins were detected in total, and 730 proteins were differently expressed, while 5950 proteins were not changed significantly. This was mainly consistent with the transcriptomic data. The comparison between the WT and 2015KO strains showed that 257 proteins were significantly differentially expressed. The number of DEPs between the 2015KO and oeT strains was 363 (Fig. 9B). In contrast, 110 proteins were differentially expressed between the WT and oeT strains. Functional classification analysis showed that these regulated (up and down) proteins were mainly located in the plastid, cytoplasm, nucleus, and membrane systems (plasma membrane and vacuolar membrane) upon *Pt2015* knockout or overexpression (Fig. 9C–E). Particularly, the percentage of plastid proteins was 38.5% in the 2015KO and oeT comparison (Fig. 9E). KEGG pathways and protein domains were also examined to identify functional information of proteins that were regulated upon *Pt2015* knockout or overexpression. Functional enrichment analysis showed that the DEPs were involved in ubiquitin-mediated proteolysis, and inositol phosphate, nitrogen, and ribosome and glycerophospholipid metabolism (Fig. 10A). Moreover, the most highly represented protein domains were a DnaJ C terminal domain, ABC (ATP-binding cassette) transporter, cyclin C (N)-terminal domain, and RNA recognition motif (Fig. 10B). Similar results were obtained when considering BP, CC, and MF (Additional file 1: Fig. S10 and S11) in the pairwise comparisons. Overall, many proteins involving different BPs changed significantly in the triradiate morphotype (oeT) that was obtained from *Pt2015* overexpression.

## Discussion

*Pt2015* is a novel gene in *P. tricornutum*, which has not been reported previously. As shown in Fig. 1 and Additional file 1: Fig. S3, Pt2015 homologs were only found in marine algae, mainly including diatoms, chrysophyta, dinoflagellates, and other red-tide algae, but not in the green lineage (*Chlamydomonas* and *Arabidopsis*). Pt2015 is localized to the plastid (Fig. 2), which is highly homologous to part of the sequences of exosome component 10 in the unicellular red algae *Porphyridium purpureum* (Additional file 1: Fig. S3B). To understand the role of the Pt2015 protein in *P. tricornutum*, *Pt2015* knockout and overexpression strains were constructed. Interestingly, the Pt2015 overexpression strain (oeT) displayed a triradiate morphotype, but the *Pt2015* knockout did not affect the morphotype (Fig. 4). Therefore, we believe that *Pt2015* positively participates in the fusiform to triradiate morphotype transformation.

To understand the pleomorphic characteristic of *P. tricornutum*, studies have reported comparative structural and physiological characteristics, and transcriptomes of fusiform, triradiate, and oval *P. tricornutum* morphotypes [28–30, 33, 52]. Nevertheless, the triradiate and oval cells used in those studies were induced or isolated from different culture conditions, of which the triradiate morphotype, particularly, was not stable. For example, the triradiate strain isolated from a natural condition was very sensitive to low salinities and low temperatures [30]. In contrast, in this study, the oeT strain was stable and has been cultured for more than 200 generations, which makes it insensitive to those conditions (Additional file 1: Fig. S6B, C, and D). The abundance of triradiate cells in the oeT was approximately 95%, which was much higher than the abundance of triradiate cells in the natural triradiate strain (approximately 70%) [32] and the induced triradiate strain (approximately 81%) [28, 33]. These data suggested that the oeT strain obtained from gene manipulation was different from the triradiate strains induced from specific culture conditions. In addition, to gain insight into the molecular characteristics of the oeT strain, transcriptomic and proteomics analysis of the WT, 2015KO, and oeT strains were performed. Both suggested that DEGs and proteins were mainly related to plastids, membrane systems, signaling pathway, and transporters (Figs. 8, 9, and 10). Additionally, the metabolic network of *Pt2015* and top 30 genes that related closely to *Pt2015* indicated that several genes (ID: 7202857, 7198159, 7197826, 7201627, 7196383, and 7203428) encoded solute carriers, channels, and transporters (Additional file 1: Fig. S13), which play an important role in cell osmoregulation, cell homeostasis, and shape [54]. Furthermore, the 2015KO strain showed a fusiform morphotype like the WT, further demonstrating that the Pt2015 protein might be a positive trigger factor during the transform from fusiform to triradiate morphotype. The Pt2015 protein might affect a series of metabolic processes in *P. tricornutum*; however, the molecular mechanism of Pt2015 in the fusiform to triradiate morphotype transformation still needs further study.

To date, microzooplankton contamination has been a major constraint in *P. tricornutum* industrial-scale cultivation, which greatly impedes the course of biomass production industrialization [16, 17, 21]. Although many strategies, including physical methods (e.g., electrical field and ultrasonication) [54, 55] and chemical methods (e.g., rotenone and quinine sulfate) [20, 56], have been trialed, they were costly and impracticable on an industrial scale. In this case, selection of microalgal strains with resistance to microzooplanktons is considered as the most practicable approach [22, 57]. In fact, changing in morphological features, such as size, cell shape, or geometry of phytoplankton, is the most obvious way to reduce grazing pressure from zooplankton in natural ecosystems [23, 24, 27]. Generally, zooplankton have two feeding modes: using mouthparts and endocytosis [27, 58]. The fusiform cells of *P. tricornutum* might be eaten by zooplankton with small mouthparts at the right angle; however, the triradiate cells with special geometry can avoid being eaten. During the co-culture of *P. tricornutum* and amoebae, the cell concentrations of oeT strain were significantly higher than that of WT (Fig. 7 and Additional file 1: Fig. S11), suggesting that the triradiate cells (the oeT strain) significantly decreased the grazing rate of the amoebae in comparison with that cultured in WT. Even if the triradiate cells were eaten through larger mouthparts or endocytosis of zooplanktons, the geometry and larger spatial structure of the triradiate cells can not only reduce grazing rates but also adversely affect membrane systems and digestion of zooplankton. Therefore, the triradiate cells of *P. tricornutum* may be the best morphotype to withstand grazing pressure in comparison with WT during industrial-scale cultivations.

The oeT strain not only displayed a stable triradiate morphotype but also accumulated larger lipids droplets than those of the WT and 2015KO strains (Figs. 4 and 6). These characteristics of the oeT strain were like the triradiate strains obtained from specific culture conditions that seemed to possess bigger lipids droplets [33]. In many algal species, an increase in lipid content was often observed during the stationary phase [11]. During this period, the growth rate of cells decreased, and nutrients start to become limited. In this study, the growth rate of the oeT strain was like that of the WT (Fig. 6C), but the lipid content in the oeT strain increased by approximately 30% compared to that of the WT, suggesting that the oeT strain could grow normally and simultaneously accumulate lipids. The transcriptomic and proteomic data demonstrated that the putative proteins (acetyl-CoA carboxylase, aldehyde dehydrogenase, and biotin synthase, etc.) involved lipid metabolism were upregulated in the oeT strain. Furthermore, the heatmap of WT-vs-oeT involving lipid metabolism also suggested that the expression of genes encoding phospholipid synthase and lipid-phosphate phosphatase were upregulated (Additional file 1: Fig. S14). In addition, the GO enrichment analysis of our proteomic data indicated that the lipid metabolisms, including sphingolipid metabolic process (GO:0006665), glycerolipid biosynthetic process (GO:0045017), membrane lipid metabolic process (GO:0006643), and glycerolipid metabolic process (GO:0046486), were upregulated in the oeT strain in comparison with WT (Additional file 1: Fig. S15). All these results explained why the lipid content in the oeT strain was higher than that of the WT.

## Conclusions

Overall, the oeT strain obtained from the overexpression of *Pt2015* was a stable *P. tricornutum* triradiate strain with a similar growth rate to the WT and increased lipid accumulation. Due to its specific geometry, the oeT strain can withstand grazing pressure during the co-culture of *P. tricornutum* and amoebae. Therefore, the oeT strain not only advances our understanding of morphotype conversion in diatoms but also demonstrates its potential applications for biofuel production.

## Supplementary Information

**Additional file 1: Figure S1.** In silico analysis of the *Phaeodactylum tricornutum Pt2015* gene*.* (A) Schematic drawing of the *Pt2015* gene on chromosome 12, which is annotated as an open reading frame of 444 bp in length corresponding to a predicted protein of 147 amino acids. (B) Analysis of the signal peptide in the Pt2015 protein using SignalP 4.1. The cleavage site of the signal peptide in Pt2015 is between the 17th and 18th amino acid. **Figure S2.** Transmembrane domain analysis of the Pt2015 protein via the TMHMM Server 2.0. **Figure S3.** (A) Comparison of the conserved regions in Pt2015 proteins from diatoms, chrysophyta, dinoflagellate, and other red-tide algae using the MEGA 7.0 platform. Conserved motifs are indicated as sequence logos, which are on the top of the sequences. (B) Amino acid sequence alignment of the Pt2015 protein with exosome component 10 in *Porphyridium purpureum*. The sequences are aligned using BioEdit. Black-boxed and gray-boxed letters represent identical or similar residues, respectively. **Figure S4.** Chloroplast purification using a discontinuous Percoll gradient (10%, 20%, and 30%) and ultracentrifugation. The chloroplast band is indicated on the right. **Figure S5.** Blue native-polyacrylamide gel electrophoresis (BN-PAGE) analysis of the thylakoid membrane complex from *P. tricornutum* wild type (WT). (A) The gel is stained with Coomassie Brilliant Blue. (B) Thylakoid membrane complexes separated by BN-PAGE in A have been further subjected to sodium dodecyl sulfate (SDS)-PAGE, and the proteins are detected with specific antibodies against PsbD, PsaA, and Pt2015, respectively. **Figure S6.** Effects of salinity and temperature on the *Pt2015* overexpression (oeT) strain. Percentage of triradiate abundance (Black square) and fusiform (Red circle) morphotypes of the oeT strain at different salinities and temperatures. (A) 100% sea water and 20 °C; (B) 50% sea water and 20 °C; (C) 30% sea water and 20 °C; and (D) 100% sea water and 10 °C. Data represent the mean ± standard deviation (SD) of four biological replicates. **Figure S7.** Validation of RNA-Sequencing data using qualitative reverse transcriptase-polymerase chain reaction (qRT–PCR) analysis. The expression of eight genes selected randomly from the list of differently expressed genes (DEGs) is shown, including ID 7,199,712 (A), 7,203,450 (B), 7,195,163 (C), 7,195,518 (D), 7,198,479 (E), 7,204,536 (F), 7,205,131 (G), and 7,198,653 (H). **Figure S8.** Gene ontology (GO) enrichment analysis (biological processes (A and B) and molecular functions (C and D)) of the wild-type (WT)-vs-*Pt2015* knockout strain (2015KO) comparison (A and C) and the WT-vs-*Pt2015* overexpression (oeT) strain comparison (B and D) based on RNA-Sequencing data. **Figure S9.** Kyoto Encyclopedia of Genes and Genomes (KEGG) enrichment analysis of the wild-type (WT)-vs-*Pt2015* knockout strain (2015KO) comparison (A) and the WT-vs-*Pt2015* overexpression (oeT) strain comparison (B) based on RNA-Sequencing data. **Figure S10.** Functional profiling analysis [biological process (A) and cellular component (B)] of the pairwise comparison of the wild type (WT), *Pt2015* knockout strain (2015KO), and *Pt2015* overexpression (oeT) strain based on proteomic data. **Figure S11.** Functional profiling analysis (molecular function) of the pairwise comparison of the wild type (WT), *Pt2015* knockout strain (2015KO), and *Pt2015* overexpression (oeT) strains based on proteomic data. **Figure S12.** Micrographs of the amoebae cultivated with the oeT (A) and WT (B) cells. The spores of amoebae were centrifugated at 3500 × *g* for 5 min, and then the precipitation was diluted with WT and oeT cells at the same cell density (5 × 10^6^ cells/ml), respectively. The diluent was cultured in culture plates (6 wells) at 25 °C under the dark condition. After 24 h, the cells of the oeT and WT were observed. The scale bar is 5 μm. **Figure S13.** The metabolic network of *Pt2015* and top 30 genes that related closely to *Pt2015* indicated that several genes encoding solute carriers or channels (ID: 7,202,857, 7,198,159, 7,197,826) and transporters (ID: 7,201,627, 7,196,383 7,203,428) based on RNA-Sequencing data. The green circle represents the *Pt2015* and the purple circles represents the genes related closely to *Pt2105*. The red lines and blues line represent positive correlation and negative correlation, respectively. **Figure S14.** The heatmap of genes involving lipid metabolisms in the comparison of WT and oeT based on RNA-Sequencing data. **Figure S15.** The GO enrichment analysis (the upregulated processes) of the oeT strain-vs-WT based on proteomic data. In the biological process, the GO ID of sphingolipid metabolic process, glycerolipid biosynthetic process, membrane lipid metabolic process, and glycerolipid metabolic process is GO:0,006,665, GO:0,045,017, GO:0,006,643, GO:0,046,486, respectively. **Table S1.** Sequences of gRNAs targeting the *Pt2015* gene in *P. tricornutum*. The predicted gRNA binding site is the underlined sequence, and the complementary oligonucleotide is also shown in the table. Oligonucleotides are designed with a top strand 5’-TCGA-3’ and bottom strand 3’-CAAA-5’ *Bsa*I restriction cut site overhangs to facilitate cloning into the Cas9 vector. **Table S2.** Lists of *Pt2015* gene and reference gene (RPS) primers.
