# Supplementary material for: Overexpression of a novel gene (Pt2015) endows the commercial diatom Phaeodactylum tricornutum high lipid content and grazing resistance
Source: Biotechnol Biofuels Bioprod. 2022 Nov 26;15:131. doi: 10.1186/s13068-022-02221-y (PMC9701398; doi:10.1186/s13068-022-02221-y)
Supplement: Supplementary file 1 — Additional file 1: Figure S1. In silico analysis of the Phaeodactylum tricornutum Pt2015 gene. (A) Schematic drawing of the Pt2015 gene on chromosome 12, which is annotated as an open reading frame of 444 bp in length corresponding to a predicted protein of 147 amino acids. (B) Analysis of the signal peptide in the Pt2015 protein using SignalP 4.1. The cleavage site of the signal peptide in Pt2015 is between the 17th and 18th amino acid. Figure S2. Transmembrane domain analysis of the Pt2015 protein via the TMHMM Server 2.0. Figure S3. (A) Comparison of the conserved regions in Pt2015 proteins from diatoms, chrysophyta, dinoflagellate, and other red-tide algae using the MEGA 7.0 platform. Conserved motifs are indicated as sequence logos, which are on the top of the sequences. (B) Amino acid sequence alignment of the Pt2015 protein with exosome component 10 in Porphyridium purpureum. The sequences are aligned using BioEdit. Black-boxed and gray-boxed letters represent identical or similar residues, respectively. Figure S4. Chloroplast purification using a discontinuous Percoll gradient (10%, 20%, and 30%) and ultracentrifugation. The chloroplast band is indicated on the right. Figure S5. Blue native-polyacrylamide gel electrophoresis (BN-PAGE) analysis of the thylakoid membrane complex from P. tricornutum wild type (WT). (A) The gel is stained with Coomassie Brilliant Blue. (B) Thylakoid membrane complexes separated by BN-PAGE in A have been further subjected to sodium dodecyl sulfate (SDS)-PAGE, and the proteins are detected with specific antibodies against PsbD, PsaA, and Pt2015, respectively. Figure S6. Effects of salinity and temperature on the Pt2015 overexpression (oeT) strain. Percentage of triradiate abundance (Black square) and fusiform (Red circle) morphotypes of the oeT strain at different salinities and temperatures. (A) 100% sea water and 20 °C; (B) 50% sea water and 20 °C; (C) 30% sea water and 20 °C; and (D) 100% sea water and 10 °C. Data represent the [file 13068_2022_2221_MOESM1_ESM.docx]

**
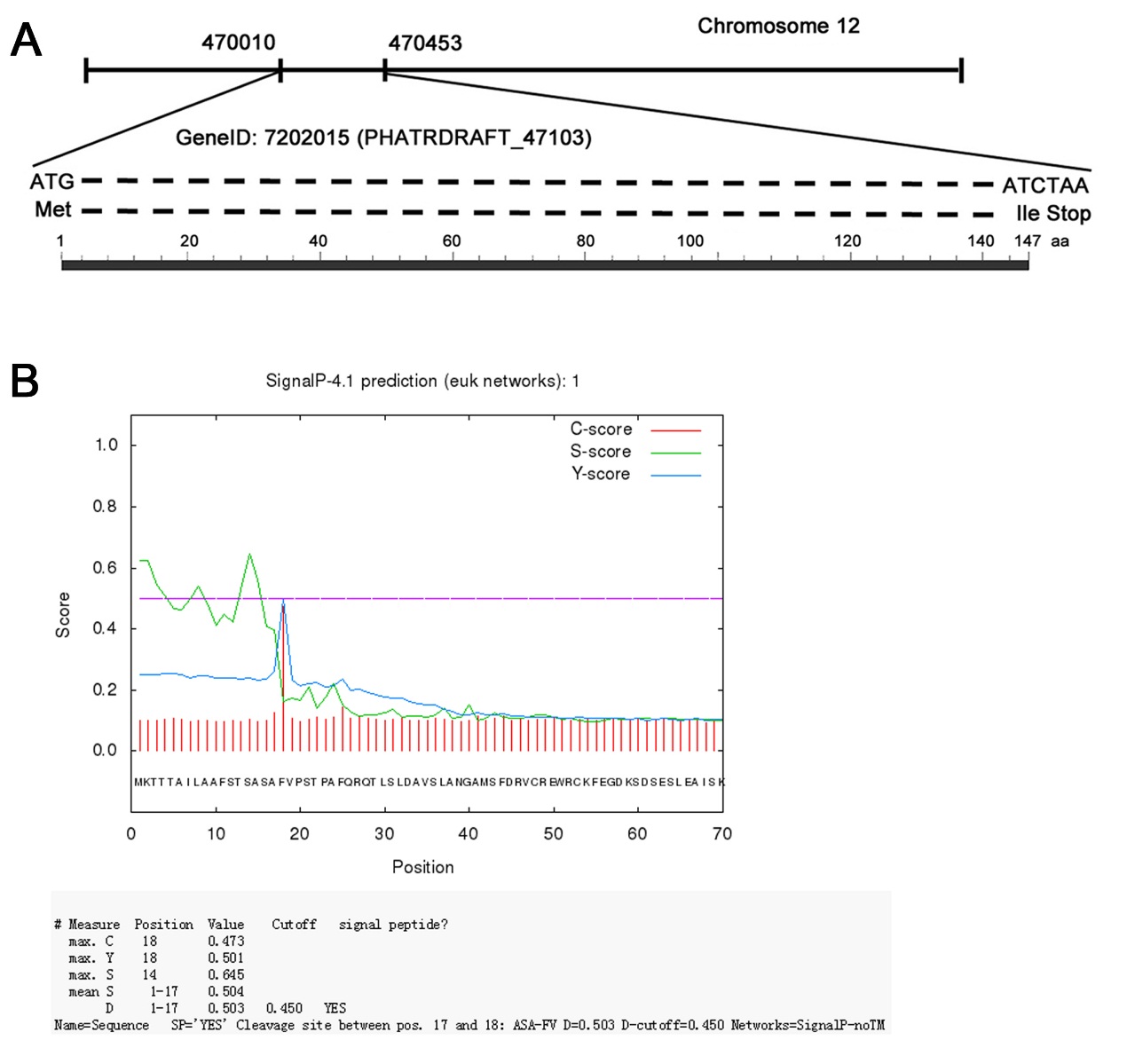
**

**Figure S1** *In silico* analysis of the *Phaeodactylum tricornutum Pt2015* gene*.* (A) Schematic drawing of the *Pt2015* gene on chromosome 12, which is annotated as an open reading frame of 444 bp in length corresponding to a predicted protein of 147 amino acids. (B) Analysis of the signal peptide in the Pt2015 protein using SignalP 4.1. The cleavage site of the signal peptide in Pt2015 is between the 17^th^ and 18^th^ amino acid.


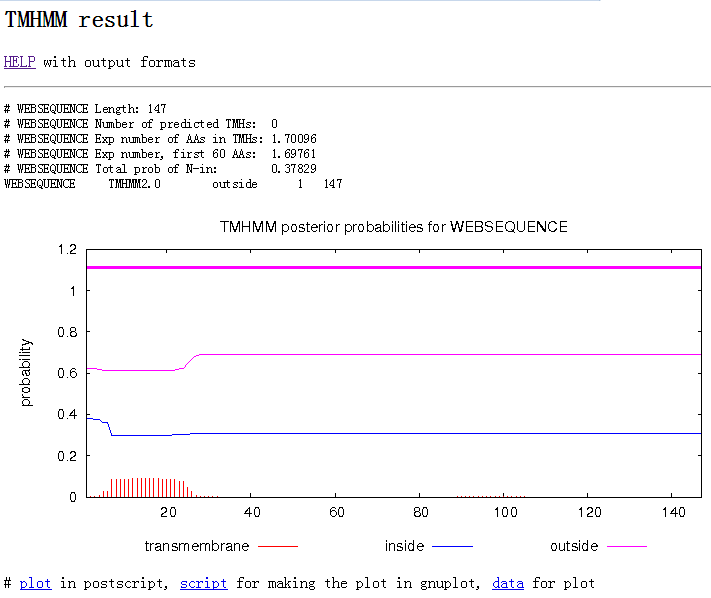


**Figure S2** Transmembrane domain analysis of the Pt2015 protein via the TMHMM Server 2.0.


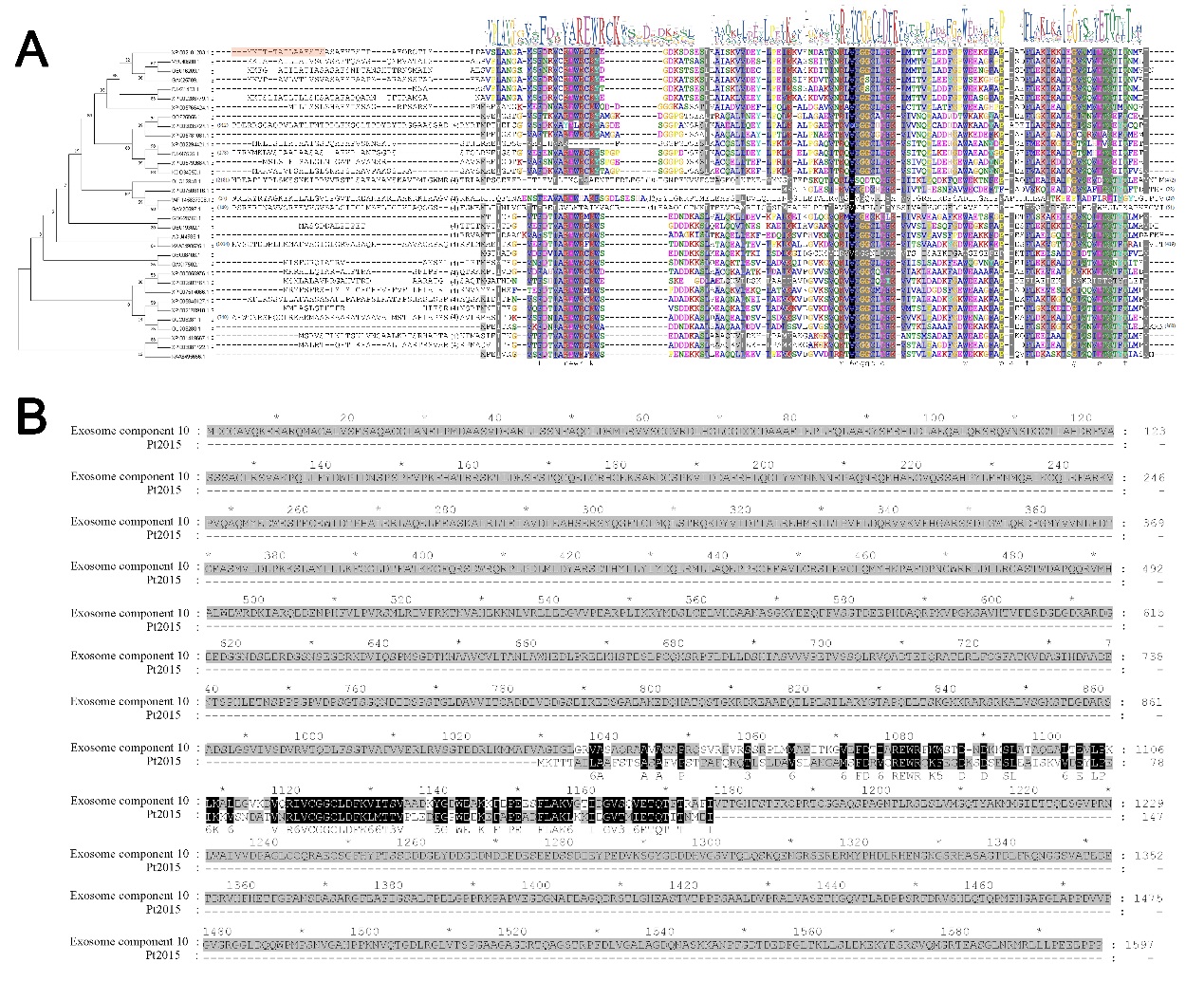


**Figure S3** (A) Comparison of the conserved regions in Pt2015 proteins from diatoms, chrysophyta, [dinoflagellate](http://dict.cn/dinoflagellate), and other red-tide algae using the MEGA 7.0 platform. Conserved motifs are indicated as sequence logos, which are on the top of the sequences. (B) Amino acid sequence alignment of the Pt2015 protein with exosome component 10 in *Porphyridium purpureum*. The sequences are aligned using BioEdit. Black boxed and gray-boxed letters represent identical or similar residues, respectively.


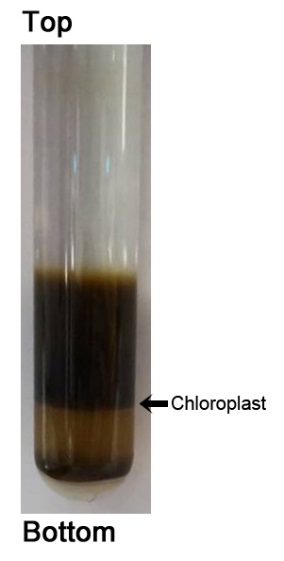


**Figure S4** Chloroplast purification using a discontinuous Percoll gradient (10%, 20%, and 30%) and ultracentrifugation. The chloroplast band is indicated on the right.


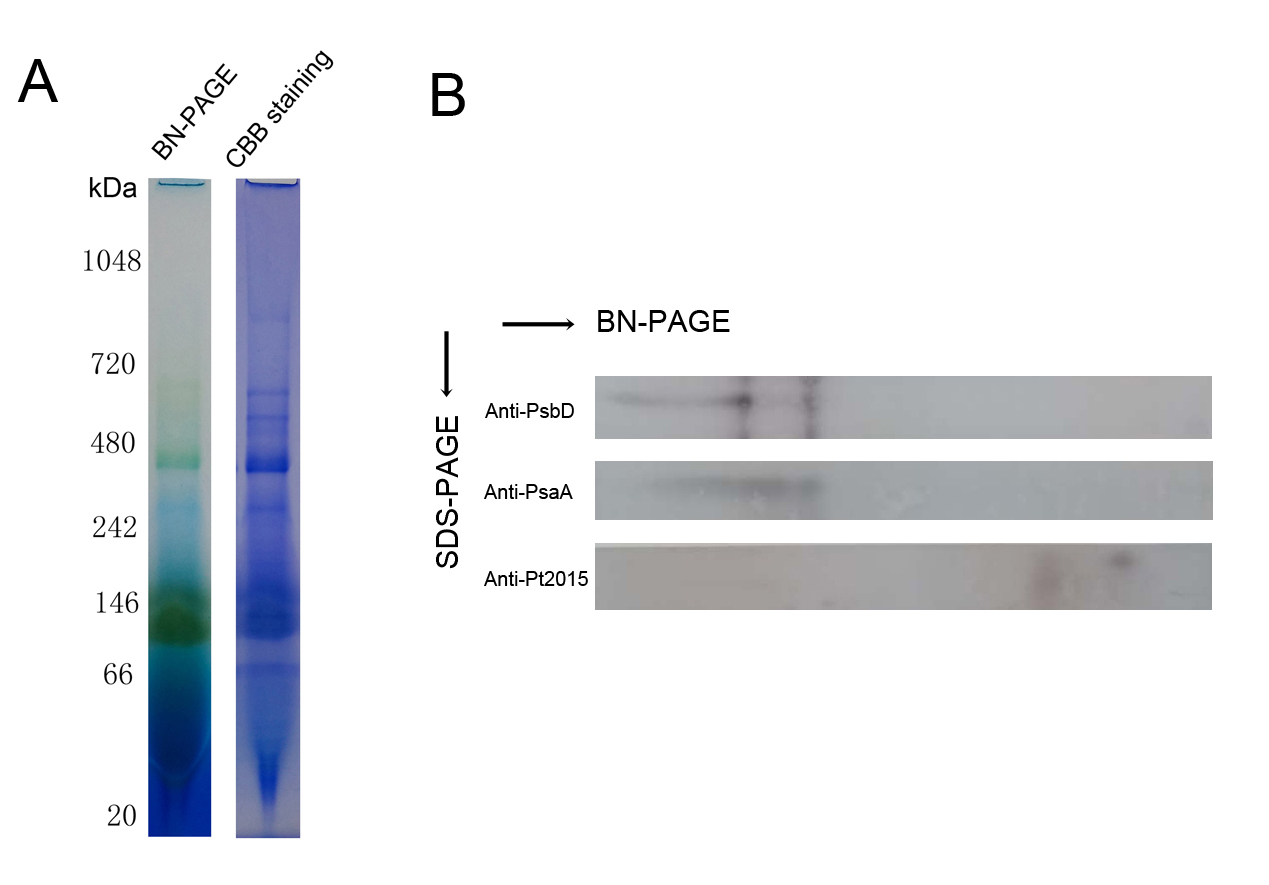


**Figure S5** Blue native-polyacrylamide gel electrophoresis (BN-PAGE) analysis of the thylakoid membrane complex from *P. tricornutum* wild type (WT). (A) The gel is stained with Coomassie Brilliant Blue. (B) Thylakoid membrane complexes separated by BN-PAGE in A have been further subjected to sodium dodecyl sulfate (SDS)-PAGE, and the proteins are detected with specific antibodies against PsbD, PsaA, and Pt2015, respectively.


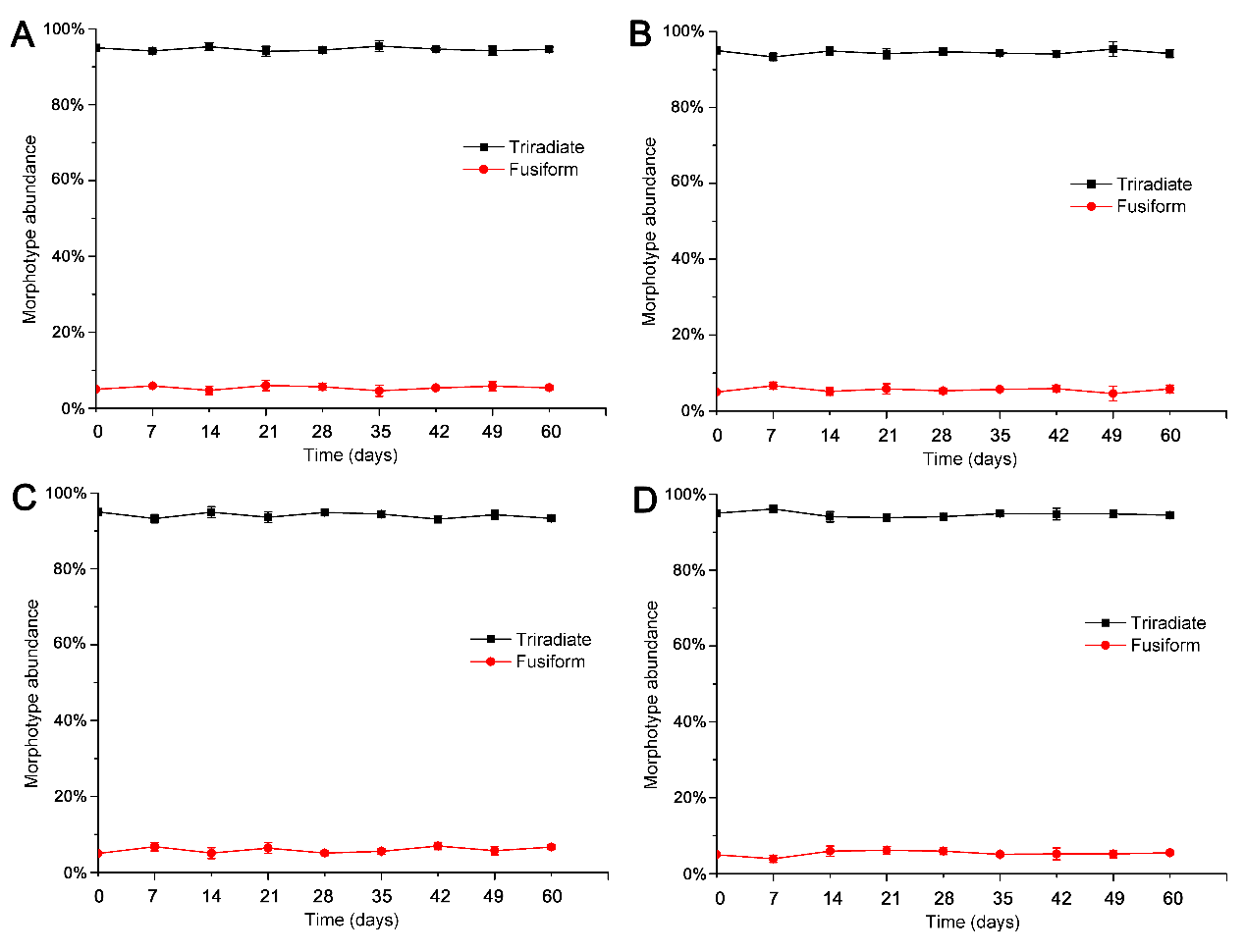


**Figure S6** Effects of salinity and temperature on the *Pt2015* over-expression (oeT) strain. Percentage of triradiate abundance (Black square) and fusiform (Red circle) morphotypes of the oeT strain at different salinities and temperatures. (A) 100% sea water and 20 °C; (B) 50% sea water and 20 °C; (C) 30% sea water and 20 °C; and (D) 100% sea water and 10 °C. Data represent the mean ± standard deviation (SD) of four biological replicates.


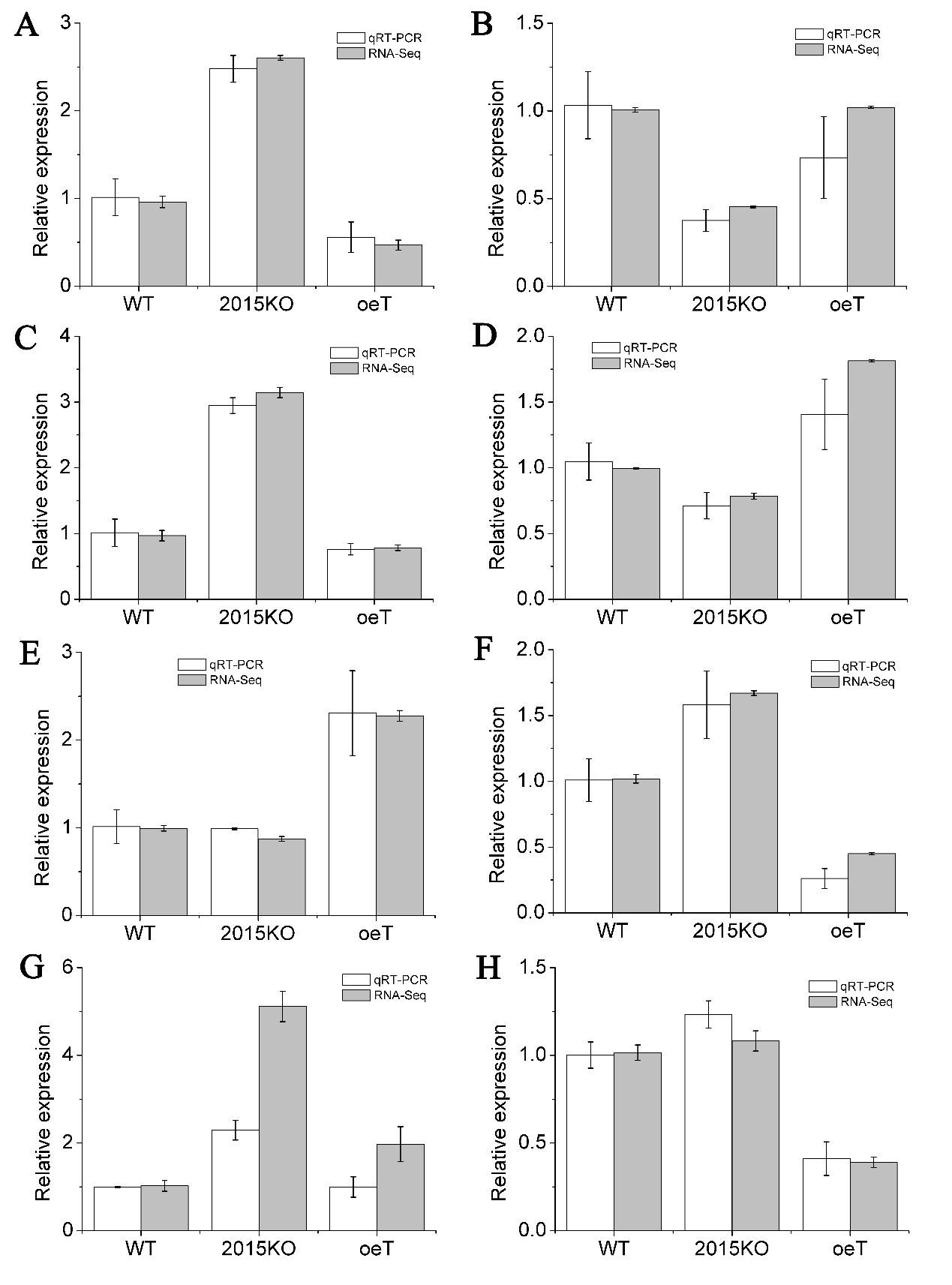


**Figure S7** Validation of RNA-Sequencing data using qualitative reverse transcriptase-polymerase chain reaction (qRT-PCR) analysis. The expression of eight genes selected randomly from the list of differently expressed genes (DEGs) is shown, including ID 7199712 (A), 7203450 (B), 7195163 (C), 7195518 (D), 7198479 (E), 7204536 (F), 7205131 (G), and 7198653 (H).


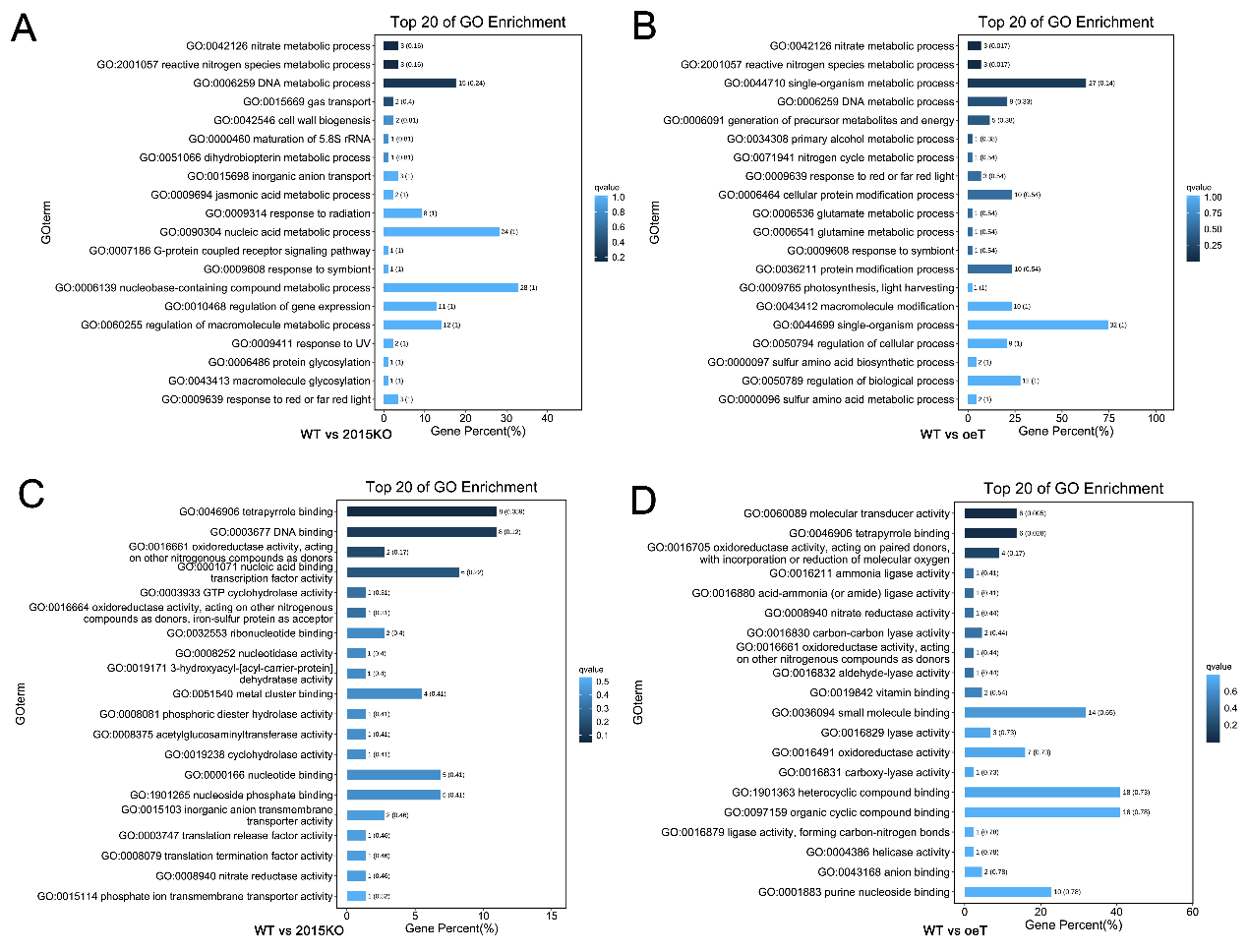


**Figure S8** Gene ontology (GO) enrichment analysis (biological processes (A and B) and molecular functions (C and D)) of the wild type (WT)-vs-*Pt2015* knockout strain (2015KO) comparison (A and C) and the WT-vs-*Pt2015* over-expression (oeT) strain comparison (B and D) based on RNA-Sequencing data.


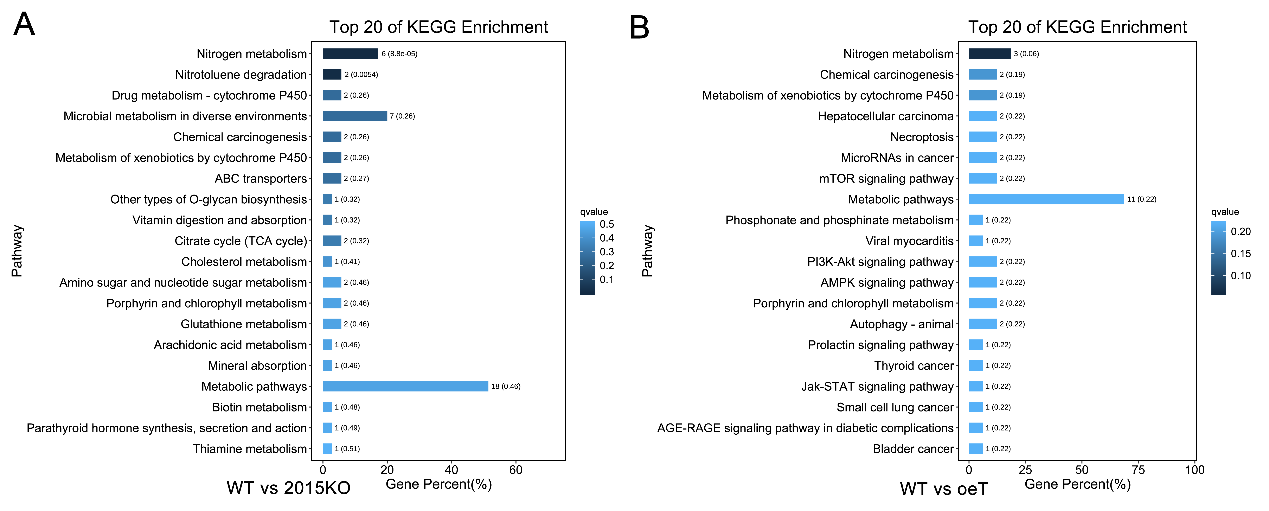


**Figure S9** Kyoto Encyclopedia of Genes and Genomes (KEGG) enrichment analysis of the wild type (WT)-vs-*Pt2015* knockout strain (2015KO) comparison (A) and the WT-vs-*Pt2015* over-expression (oeT) strain comparison (B) based on RNA-Sequencing data.

**
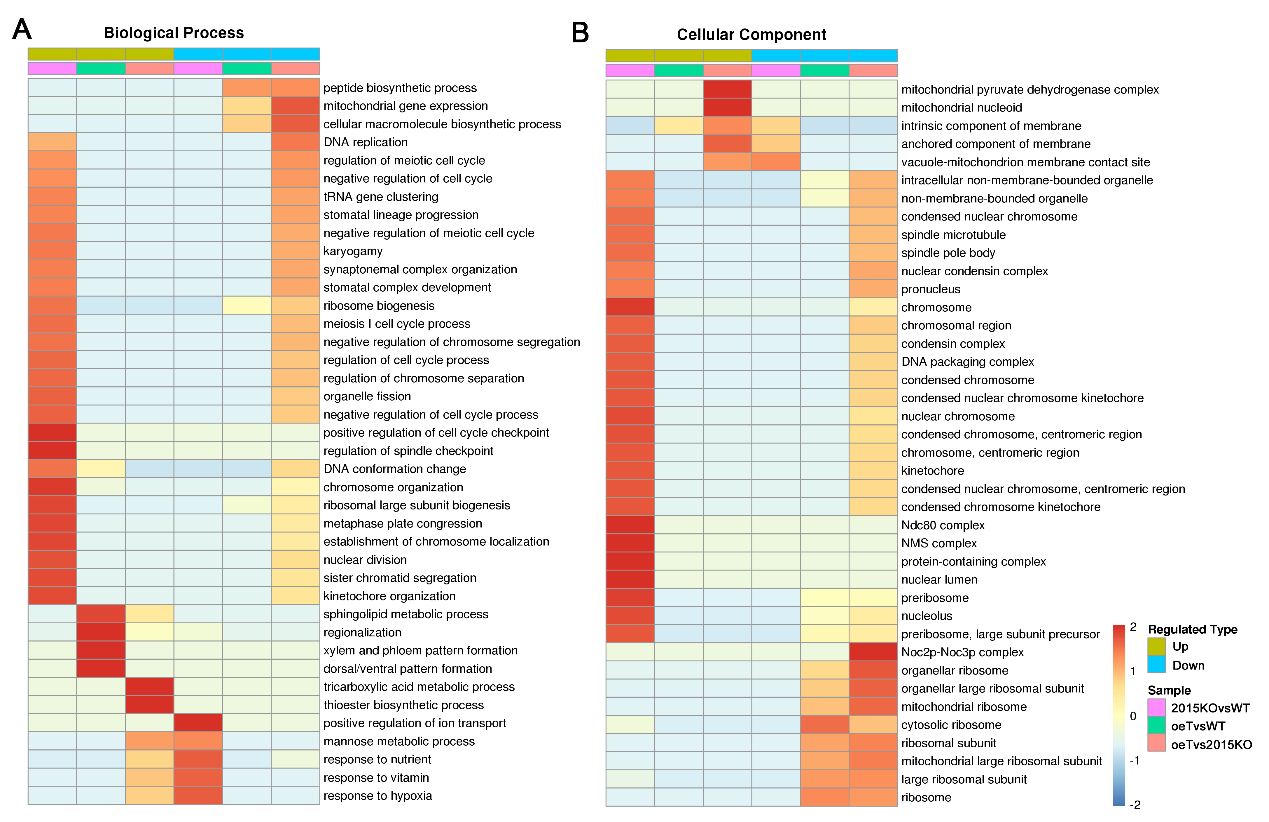
**

**Figure S10** Functional profiling analysis [biological process (A) and cellular component (B)] of the pairwise comparison of the wild type (WT), *Pt2015* knockout strain (2015KO), and *Pt2015* over-expression (oeT) strain based on proteomic data.

**
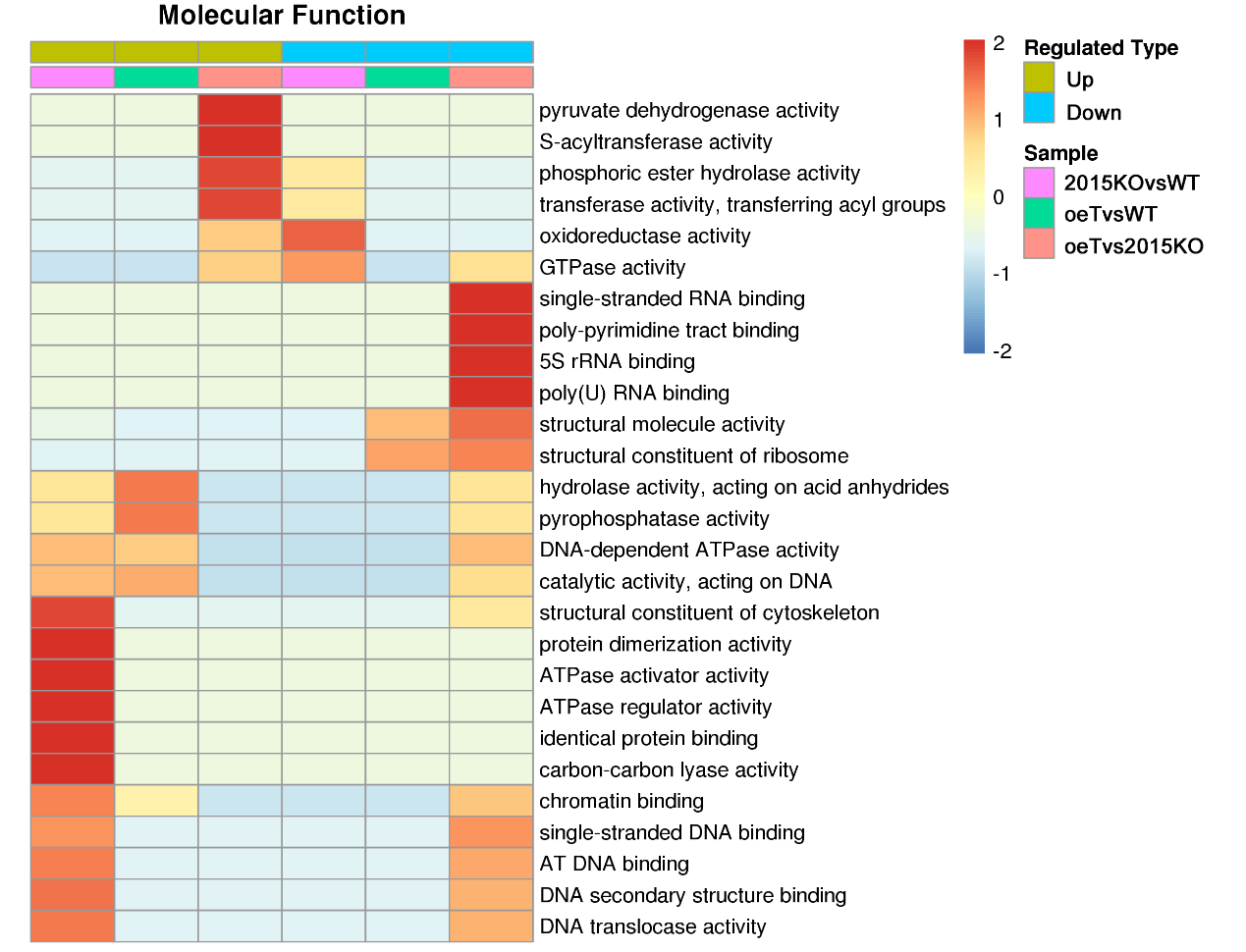
**

**Figure S11** Functional profiling analysis (molecular function) of the pairwise comparison of the wild type (WT), *Pt2015* knockout strain (2015KO), and *Pt2015* over-expression (oeT) strains based on proteomic data.


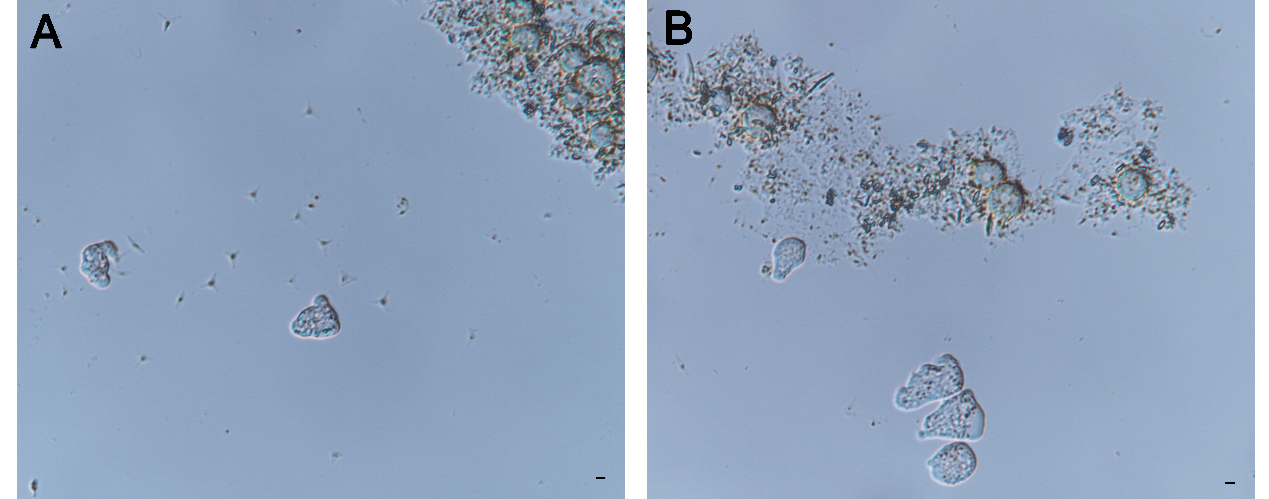


**Figure S12** Micrographs of the amoebae cultivated with the oeT (A) and WT (B) cells. The spores of amoebae were centrifugated at 3500 × *g* for 5 min, and then the precipitation were diluted with WT and oeT cells at the same cell density (5x10^6^ cells/ml), respectively. The diluent were cultured in culture plates (6 wells) at 25 °C under the dark condition. After 24 hours, the cells of the oeT and WT were observed. The scale bar is 5 μm.


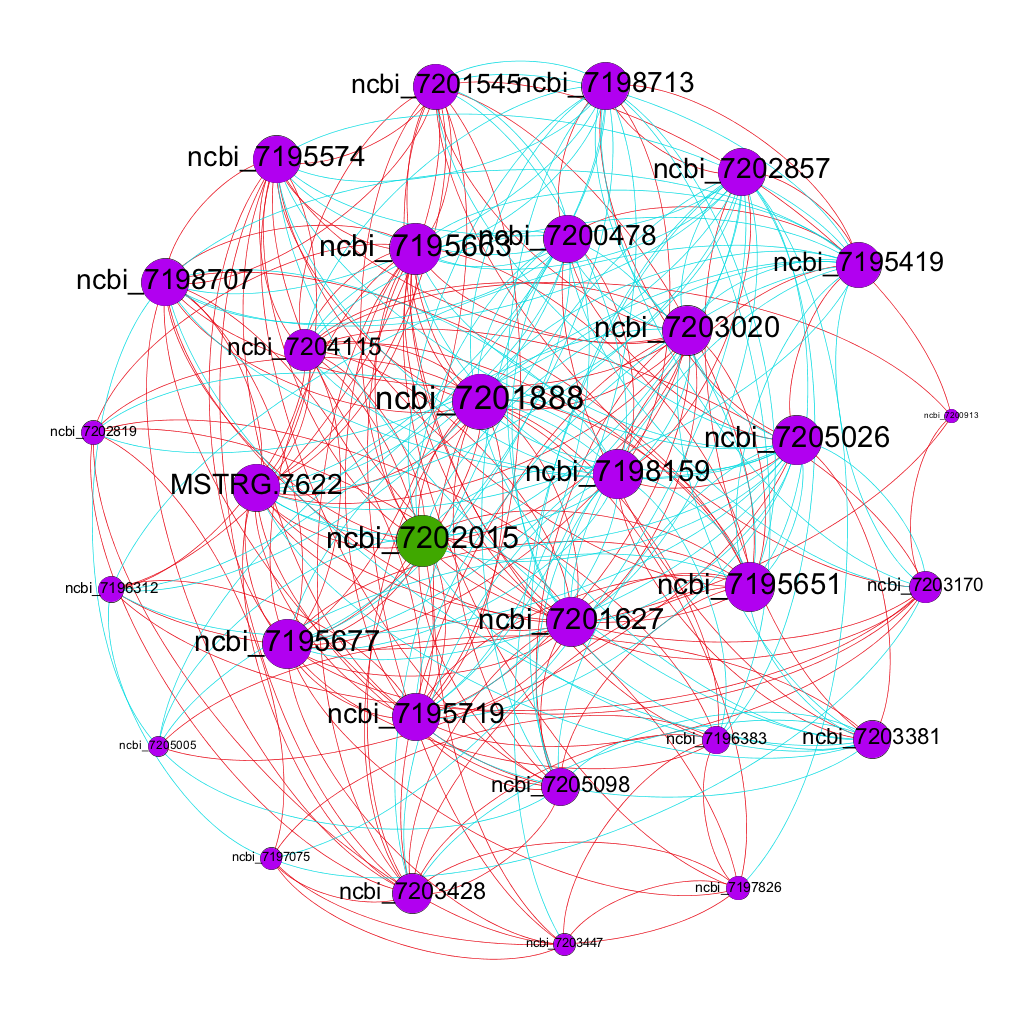


**Figure S13** The metabolic network of *Pt2015* and top 30 genes that related closely to *Pt2015* indicated that several genes encoding solute carriers or channels (ID: 7202857, 7198159, 7197826) and transporters (ID: 7201627, 7196383 7203428) based on RNA-Sequencing data. The green circle represented the *Pt2015* and the purple circles represented the genes related closely to *Pt2105*. The red lines and blues line represent positive correlation and negative correlation, respectively.


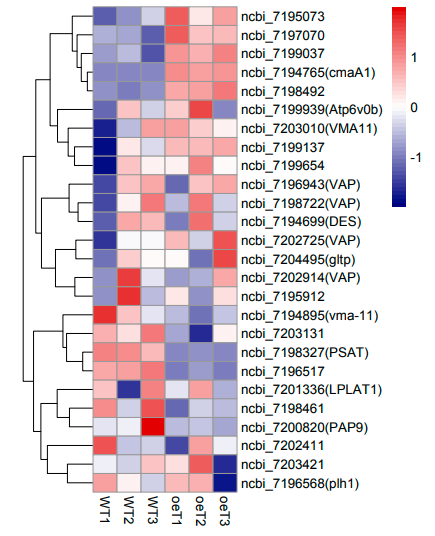


**Figure S14** The heatmap of genes involving lipid metabolisms in the comparison of WT and oeT based on RNA-Sequencing data.


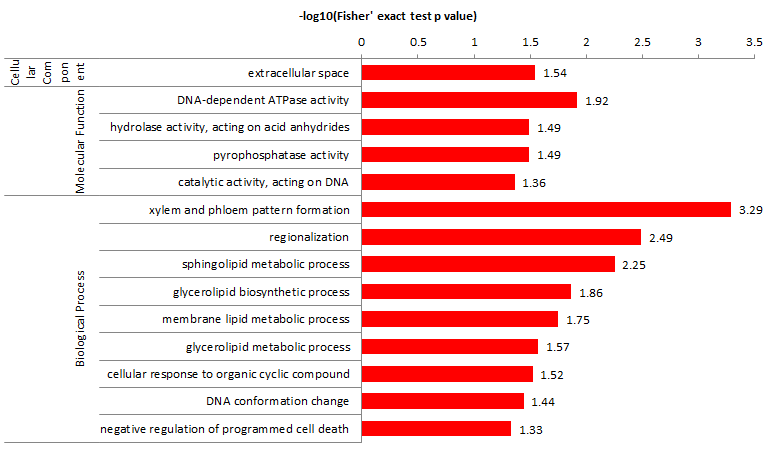


**Figure S15** The GO enrichment analysis (the up-regulated processes) of the oeT strain-vs-WT based on proteomic data. In the biological process, sphingolipid metabolic process, glycerolipid biosynthetic process, membrane lipid metabolic process and glycerolipid metabolic process is GO:0006665, GO:0045017, GO:0006643, GO:0046486, respectively.

**Table S1** Sequences of gRNAs targeting the *Pt2015* gene in *P. tricornutum*. The predicted gRNA binding site is the underlined sequence, and the complementary oligonucleotide is also shown in the table. Oligonucleotides are designed with a top strand 5’-TCGA-3’ and bottom strand 3’-CAAA-5’ *Bsa*I restriction cut site overhangs to facilitate cloning into the Cas9 vector.

| **gRNA number** | **Annealed oligos for cloning** |
| --- | --- |
| 2015gRNA1 | 5’-TCGAGATGCCGTTTCGTTAGCCAA -3’  3’-CTACGGCAAAGCAATCGGTTCAAA-5’ |
| 2015gRNA2 | 5’-TCGACCGAACAAGAGTCGAGATGG -3’  3’-GGCTTGTTCTCAGCTCTACCCAAA-5’ |
| 2015gRNA3 | 5’-TCGAGGCAAGGAAATCAGCCTCCG -3’  3’-CCGTTCCTTTAGTCGGAGGCCAAA-5’ |

**Table S2** Lists of *Pt2015* gene and reference gene (RPS) primers

| **Primer name** | | **Sequence** | **Use** |
| --- | --- | --- | --- |
| 2015gF1 | ACCGCTATTCTTGCTGCCTT | | Amplifying the on-target site |
| 2015gR1 | | TGGCAAGGAAATCAGCCTCC |  |
| 2015gF2 | | TAAGTAGACAGCAGCTGGCG | Amplifying the on-target site |
| 2015gR2 | | AAGGCAGCAAGAATAGCGGT |  |
| 2015gF3  2015gR3 | | GTGAATCGTTTGGTGTGCGG  TCGTTCGACGGCTTCTTTCA | Amplifying the on-target site |
| YzF  YzR | | GGAGGATCAGATTCAGATTACA  AGAGCAAGGATGCCCATT | Amplifying the pPha-T1 vector including 2015 CDS |
| 2015qF | | AGGTCTCCAATGATGCTACGG | RT-PCR |
| 2015qR | | TTTTCTTCCCAGGGTCCAA | RT-PCR |
| RPSqF | | CGAAGTCAACCAGGAAACCAA | RT-PCR |
| RPSqR | | GTGCAAGAGACCGGACATACC | RT-PCR |
| Pt2015oe-F | | GAATTCATGAAAACAACGACCGCT | Overexpression |
| Pt2015oe-R | | AAGCTTTTAGATTTCCATGTTGGT | Overexpression |
|  | |  |  |
| Pt2015eGFP-F | | GGGAATTCATGAAAACAACGACCGCT | Overexpression |
| Pt2015eGFP-R | | GGGGTACCTCCTCCTCCTCCTCCCCTAGGTCCGATTTCCATGTTGGTAATCGT | Overexpression |
